# Research on strengthening of T-shaped reinforced concrete simply supported beams with U-shaped jackets to enlarge the cross-section: Theoretical analysis, experiments, and finite element analysis

**DOI:** 10.1371/journal.pone.0361416

**Published:** 2026-10-27

**Authors:** Jian-fa Wang, En-he Bao, Ming-su Zhou, Yong-song Wu, Chang-jian Xiao

**Affiliations:** 1 College of Civil Engineering, Guizhou University, Guiyang, Guizhou, China; 2 Guizhou Provincial Key Laboratory of Rock and Soil Mechanics and Engineering Safety, Guiyang, Guizhou, China; 3 International College, Krirk University, Bangkok, Thailand; Universiti Teknologi Malaysia, MALAYSIA

## Abstract

To promote the reuse of existing beams, an experimental investigation was conducted on T-shaped reinforced concrete (RC) beams strengthened with U-shaped RC jackets. Three specimens were tested: one unstrengthened T-shaped control beam and two strengthened beams. Each jacket comprised additional reinforcing bars and higher-strength concrete cast along both sides of the web and the beam soffit, transforming the original T-shaped cross-section into a rectangular section. All three specimens were subjected to cyclic loading, and corresponding finite element simulations were performed to investigate their hysteretic response, skeleton response, flexural deformation, and failure modes. The strengthened beams exhibited load-carrying capacities more than twice that of the unstrengthened beam, and the finite element simulations generally reproduced the experimental responses. Based on truss–arch synergy, an analytical model was developed to predict the load-carrying capacity of RC beams. The predictions agreed well with the experimental results, supporting the reliability of the proposed model.

## 1. Introduction

Reinforced concrete (RC) T-beams are widely used in bridges and building floor systems. Owing to material degradation, reinforcement corrosion, accidental damage, and increased load demands, some existing beams can no longer meet current strength and serviceability requirements. Retrofitting can extend their service life while reducing resource consumption and environmental impacts [1]. However, the flange-web configuration affects the position of the neutral axis, the flexural compression zone, shear-transfer mechanisms, and crack-propagation paths. When the loading direction is reversed, the flange shifts from compression to tension, thereby changing the relative contributions of the original beam and the added strengthening components to load resistance. Therefore, findings obtained from rectangular beams or under monotonic loading cannot be directly applied to T-beams [2–4].

Existing methods for strengthening RC beams mainly include composite strengthening systems, thin overlays of high-performance cementitious materials, and section enlargement. Although fiber-reinforced polymer (FRP), steel-reinforced grout (SRG), and fabric-reinforced cementitious matrix (FRCM) systems offer the advantages of low weight, high strength, and rapid installation, their effectiveness is often limited by premature end debonding, concrete-cover separation, and anchorage constraints. In T-beams, the flange further limits the effective bond length available for U-wraps, whereas supplemental anchorage increases detailing and construction complexity. Moreover, improvements in strength may be accompanied by reduced ductility [5–8].

An analysis of a database comprising 410 tests showed that the wrapping configuration, material properties, shear-span ratio, and stirrup ratio of the original beam jointly govern the shear capacity and failure mode; nevertheless, predictions obtained using existing models remain inconsistent [9].

Thin layers of ultra-high-performance fiber-reinforced concrete (UHPFRC), ultra-high-strength engineered cementitious composite (UHS-ECC), and engineered geopolymer composite (EGC) can enhance crack control and compatibility with the existing concrete substrate. However, when these highly ductile matrices are used alone, the resulting improvement in load-carrying capacity is often limited; increasing the amount of composite reinforcement may, in turn, cause strain concentration and interfacial debonding [10–12]. These findings indicate that the use of advanced materials does not eliminate the fundamental challenges associated with interfacial load transfer and anchorage.

Although conventional RC jacketing increases member self-weight and cross-sectional dimensions, it also enlarges the concrete cross-sectional area, increases the effective depth, and provides additional longitudinal reinforcement and stirrups, thereby yielding relatively balanced improvements in load-carrying capacity, stiffness, ductility, fire resistance, and durability [1,13,14].

However, existing studies have primarily focused on rectangular beams strengthened with one- or three-sided RC jackets, with particular emphasis on monotonic behavior and peak load-carrying capacity. Studies on U-shaped jackets have typically employed strain-hardening cementitious composites (SHCC), ultra-high-performance concrete (UHPC), highly ductile fiber-reinforced concrete (HDC), or UHPFRC to strengthen damaged or shear-critical members. Although these systems can enhance load-carrying capacity and post-peak deformation capacity, the initial stiffness of the strengthened member may not be fully restored; moreover, pre-existing damage can further reduce strengthening effectiveness [15–19].

Studies of cyclic behavior have mainly focused on thin-layer systems based on textile-reinforced mortar (TRM) and carbon fiber-reinforced polymer (CFRP). These studies indicate that interfacial damage governs stiffness degradation and energy dissipation and that increasing the amount of external reinforcement does not necessarily produce sustained performance improvements [20,21]. Recent analyses of impact-test databases and near-field blast experiments have also shown that loading characteristics and interfacial damage can alter the failure behavior of strengthened beams [22,23]. Therefore, the cyclic behavior of T-beams strengthened with relatively thick conventional U-shaped RC jackets under both positive and negative loading remains unclear.

The interface between the existing and newly cast concrete is critical to achieving monolithic behavior in the strengthened section. Interfacial shear is transferred through adhesion, aggregate interlock, friction, and the dowel action and confinement provided by the reinforcement. Inadequate force transfer may cause interfacial slip, separation, or cracking, resulting in a transition from full to partial composite action. Studies accounting for interfacial slip indicate that the assumption of full composite action may overestimate the initial stiffness, whereas U-shaped jackets generally provide better sectional integrity than single-sided jackets because they engage the existing member along three surfaces [24].

Tests on T-shaped core beams with roughened interfaces and U-shaped jackets have shown that positive- and negative-moment regions differ in their sensitivity to interfacial slip [25]. Interface roughening and post-installed reinforcement have also been shown to help damaged T-beams maintain composite action [26]. Cohesive-zone and bond–slip models further indicate that interfacial degradation affects unloading stiffness, residual deformation, and crack propagation [27–29]. In addition, internal forces are redistributed between the stirrups in the jacket and those in the original beam, meaning that their respective contributions cannot be determined by simple summation [30]. However, experimental evidence is still lacking on how surface roughness affects the cyclic composite performance of T-beams strengthened with conventional U-shaped RC jackets and whether interfacial failure may occur.

Conventional U-shaped RC jackets offer several practical advantages, including readily available materials, manageable costs, well-established construction techniques, good fire resistance and durability, and favorable deformation compatibility with the existing concrete. They can be constructed around the beam soffit and along both sides of the web without removing the existing flange. Together, the additional longitudinal reinforcement, L-shaped anchor bars, U-shaped stirrups, and newly cast jacket concrete transform the original T-section into a rectangular composite section, establishing multiple interfacial load-transfer mechanisms through adhesion, aggregate interlock, friction, and the dowel action and confinement provided by the reinforcement.

Current research on beam strengthening has largely focused on advanced materials, whereas conventional RC jacketing has received comparatively limited attention. Studies [24] and [25] mainly examined the interfacial behavior between concrete jackets and existing beams but did not investigate analytical methods for predicting the load-carrying capacity of RC-jacketed beams. Although the applicable strengthening design code [31] provides a method for calculating the load-carrying capacity of such beams, the equations involved are relatively complex, making the calculation procedure cumbersome. Therefore, further research is needed to develop a theoretical model that provides both predictive accuracy and computational simplicity.

To address the aforementioned research gaps, cyclic loading experiments were conducted on three specimens: one unstrengthened existing T-beam and two strengthened beams with rectangular sections, each comprising an existing T-beam and an 80-mm-thick U-shaped RC jacket. The overall strengthening effectiveness of the U-shaped RC jacket and the influence of interface roughening on the mechanical behavior of the strengthened beams were preliminarily evaluated by examining the load-carrying capacity, failure modes, hysteretic response, stiffness degradation, ductility, and energy dissipation capacity of the specimens.

The load-carrying capacities of the strengthened beams were calculated using both the truss-arch model and the code-based method. The predictions were compared with the experimental results to evaluate the predictive accuracy and computational simplicity of the truss–arch model relative to the code-based method. An ABAQUS finite element model was also developed to simulate the hysteretic response and damage evolution of the specimens and was validated against the experimental results. Furthermore, parametric analyses were conducted to investigate the influence of the viscosity parameter of the concrete damaged plasticity (CDP) model on the numerical results and the effect of the diameter of the upper longitudinal reinforcing bars in the jacket on the mechanical behavior of the strengthened beams.

The main contributions of this study are threefold: (1) expanding the existing research on the cyclic behavior of T-beams strengthened with conventional U-shaped RC jackets and developing a load-carrying capacity model that combines predictive accuracy with computational simplicity, thereby providing a practical complement to code-based calculation methods; (2) developing an experimentally validated finite element model capable of reproducing the hysteretic response and damage evolution of the strengthened beams; and (3) establishing an integrated experimental–analytical–numerical evaluation framework and providing preliminary evidence of how interface roughness affects the composite action between the existing and newly cast concrete. These contributions provide a basis for interface treatment, load-carrying capacity assessment, and engineering applications of U-shaped RC jacketing.

## 2. Analytical model for the load-carrying capacity of RC beams

This section presents an analytical model for predicting the maximum load-carrying capacity of RC-jacketed beams and compares its predictions with those obtained using the method prescribed by the applicable strengthening design code.

Fig 1(a) shows an RC beam subjected to two symmetrically applied concentrated loads. The main longitudinal reinforcement (flexural reinforcement) is placed at the top and bottom of the beam, while stirrups (shear reinforcement) are distributed along the beam to resist shear.

**Fig 1:**
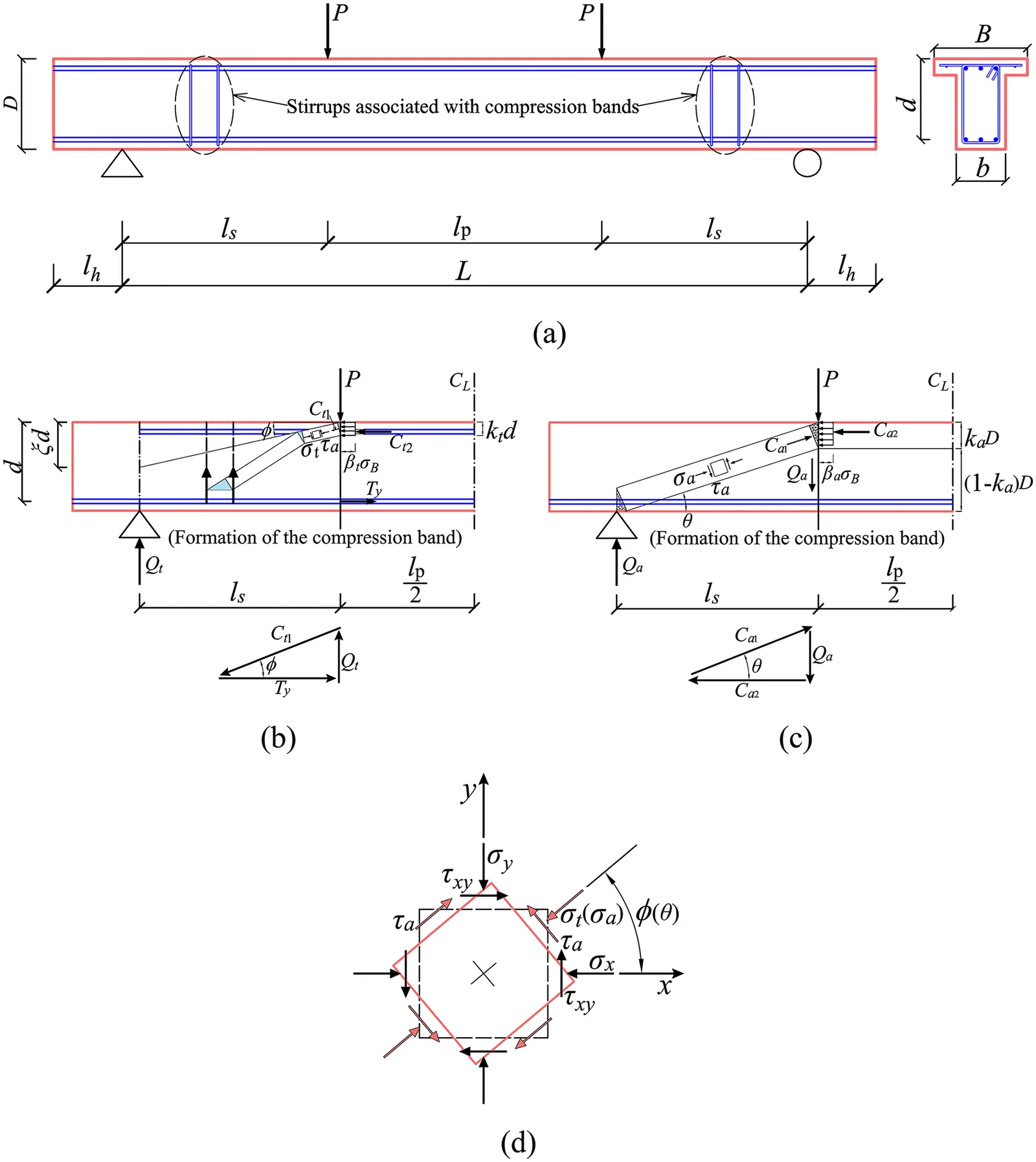
Truss and arch models of RC beams. (a) RC beam under symmetric two-point loading. (b) Truss action. (c) Arch action. (d) Stress element in Cartesian coordinates.

### 2.1 Truss action

Reference [32] indicates that three inclined concrete compression bands develop within each shear span between a concentrated load and the adjacent support, as shown in Fig 1(b). The angle *ϕ* denotes the inclination of the compression band adjacent to the loading point relative to the compression face of the beam. The vertical distance from the compression face to the intersection of the extended centerline of this band with the support line is denoted by *ξd*, where *ξ* is a dimensionless coefficient and *d* is the effective depth of the beam. The compressive force *C*_*t*1_ in this band, the yield tensile force *T*_*y*_ in the longitudinal flexural reinforcement, and the support reaction *Q*_*t*_, which equals the shear force in the shear span, satisfy force equilibrium and collectively form the truss action. In addition, a horizontal compression band develops near the top of the beam within the pure-flexure region between the two loading points, connecting the inclined compression bands in the two shear spans.

In the inclined coordinate system of a compression band oriented at an angle *ϕ*, the plane-stress state comprises a normal compressive stress *σ*_*t*_ and a shear stress *τ*_*t*_, with *τ*_*t*_ assumed to be zero. Resolving this stress state into the horizontal and vertical directions yields the normal stresses *σ*_*x*_ and *σ*_*y*_ and the shear stress *τ*_*xy*_, thereby defining an orthogonal stress field. The corresponding infinitesimal stress element in the Cartesian coordinate system is shown in Fig 1(d). Assuming that *σ*_*x*_ = *β*_*t*_*σ*_*B*_, the shear stress *τ*_*xy*_ can be derived as follows:


$$\begin{array}{c} \tau_{xy}\;=\;\beta_{t}\sigma_{B}\text{tan}\phi \end{array}$$ (1)

In Eq 1, *β*_*t*_ is the compressive stress coefficient, and *σ*_*B*_ denotes the compressive strength of concrete. Force equilibrium requires the resultant compressive force *C*_*t*2_ to equal the tensile force *T*_*y*_ in the longitudinal flexural reinforcement at yielding, i.e., *C*_*t*2_ = *T*_*y*_. Substituting *C*_*t*2_ = *bk*_*t*_*dβ*_*t*_*σ*_*B*_ yields the following equilibrium equation:


$$\begin{array}{c} bk_{t}d\beta_{t}\sigma_{B}\text{\textasciitilde{}}=\;a_{t}\sigma_{y} \end{array}$$ (2)

In Eq 2, *k*_*t*_ is the compression-zone depth coefficient, *b* is the beam width, *a*_*t*_ is the cross-sectional area of the longitudinal flexural reinforcement, and *σ*_*y*_ is the yield strength of that reinforcement. Accordingly, the load-carrying capacity *Q*_*t*_ provided by the truss action can be expressed as follows:


$$\begin{array}{c} Q_{t}\text{\textasciitilde{}}=\;\tau_{xy}bk_{t}d \end{array}$$ (3)

Substituting Eq 1 into Eq 3 and combining the result with Eq 2 and the geometric relationship tan*ϕ* = *ξd*/*l*_*s*_ yields the following expression for *Q*_*t*_:


$$\begin{array}{c} Q_{t}\text{\textasciitilde{}}=\;\xi a_{t}\sigma_{y}\frac{\text{1}}{\lambda_{d}} \end{array}$$ (4)

Here, *λ*_*d*_ denotes the shear-span-to-effective-depth ratio, defined as *λ*_*d*_ = *l*_*s*_/*d*, where *l*_*s*_ is the shear span. Accordingly, the bending moment *M*_*t*_ associated with the truss action, given by *M*_*t*_ = *Q*_*t*_*l*_*s*_, can be expressed as follows:


$$\begin{array}{c} M_{t}\text{\textasciitilde{}}=\;\xi a_{t}\sigma_{y}d \end{array}$$ (5)

In this equation, *ξ* is a geometric coefficient used to determine the inclination angle *ϕ* of the inclined compression band in the truss mechanism. Because this study adopts the truss-action model presented in Reference [32], the recommended value *ξ* = 0.9 is used. This value yields the maximum calculated load-carrying capacity attributable to the truss action and is adopted directly from Reference [32], rather than fitted to the experimental results obtained in this study.

### 2.2 Arch action

The compressive stress *β*_*t*_*σ*_*B*_ mobilized by the truss action is lower than the effective concrete compressive strength *β*_*c*_*σ*_*B*_ The remaining compressive stress available for the arch action is therefore expressed as *β*_*a*_*σ*_*B*_ = *β*_*c*_*σ*_*B*_-*β*_*t*_*σ*_*B*_. As shown in Fig 1(c), *θ* denotes the angle between the inclined compression band in the flexure-shear region and the horizontal axis. The inclined compression bands in the shear spans on both sides of the beam connect to the horizontal compression band between the loading points, collectively forming a complete trapezoidal arch mechanism.

The stress components *σ*_*a*_ and *τ*_*a*_ acting on the inclined compression band are related to the components *σ*_*x*_, *σ*_*y*_, and *τ*_*xy*_ in the Cartesian coordinate system through the following stress-transformation equation:


$$\begin{array}{c} \sigma_{x}\text{\textasciitilde{}}=\;\sigma_{a}{\text{cos}}^{\text{2}}\phi \text{+2}\tau_{a}\text{sin}\phi \cdot \text{cos}\phi \end{array}$$ (6)

$$\begin{array}{c} \sigma_{y}\text{\textasciitilde{}}=\;\sigma_{a}{\text{sin}}^{\text{2}}\phi \text{-2}\tau_{a}\text{sin}\phi \cdot \text{cos}\phi \end{array}$$ (7)

$$\begin{array}{c} \sigma_{a}\text{\textasciitilde{}}=\;\sigma_{x}\text{+}\sigma_{y} \end{array}$$ (8)

$$\begin{array}{c} \tau_{xy}\text{\textasciitilde{}}=\;\sigma_{a}\text{sin}\phi \cdot \text{cos}\phi \text{-}\tau_{a}\left({\text{cos}}^{\text{2}}\phi {\text{-sin}}^{\text{2}}\phi \right) \end{array}$$ (9)

From the stress transformation shown in Fig 1(d), the shear stress *τ*_*xy*_ can be expressed as follows:


$$\begin{array}{c} \tau_{xy}\text{\textasciitilde{}}=\;\frac{\text{1}}{\text{2}}\beta_{a}\sigma_{B}\text{tan}\theta \end{array}$$ (10)

The load-carrying capacity *Q*_*a*_ provided by the arch action can be expressed as follows:


$$\begin{array}{c} Q_{a}\text{\textasciitilde{}}=\;\tau_{xy}bk_{a}D \end{array}$$ (11)

Substituting Eq 10 into Eq 11 and applying the geometric relationship tan*θ*=(1-*k*_*a*_)*D/l*_*s*_ yields the following expression:


$$\begin{array}{c} Q_{a}\text{\textasciitilde{}}=\;\frac{\text{1}}{\text{2}}\beta_{a}\sigma_{B}b\frac{k_{a}\text{(1}-k_{a}\text{)}D^{\text{2}}}{l_{s}} \end{array}$$ (12)

The coefficient *k*_*a*_ is defined as the ratio of the width of the inclined compression band to the overall beam depth *D*. According to Eq 12, *Q*_*a*_ reaches its maximum at *k*_*a*_ = 0.5. Therefore, the maximum load-carrying capacity provided by the arch action can be expressed as follows:


$$\begin{array}{c} Q_{a}\text{\textasciitilde{}}=\;\frac{\text{1}}{\text{8}}\beta_{a}\sigma_{B}b\frac{D}{\lambda_{D}} \end{array}$$ (13)

In Eq 13, *λ*_*D*_ denotes the shear-span-to-overall-depth ratio, defined as *λ*_*D*_ = *l*_*s*_/*D*, where *D* is the overall beam depth. Accordingly, the bending moment *M*_*a*_ associated with the arch action, given by *M*_*a*_ = *Q*_*a*_*l*_*s*_, can be expressed as follows:


$$\begin{array}{c} M_{a}\text{\textasciitilde{}}=\;\frac{\text{1}}{\text{8}}\beta_{a}\sigma_{B}bD^{\text{2}} \end{array}$$ (14)

In this equation, *β*_*a*_ = *β*_*c*_-*β*_*t*_ is the concrete compressive stress coefficient associated with the arch action. According to Reference [32], the calculated load-carrying capacity provided by the arch action reaches its maximum at *β*_*a*_ = 0.15. Therefore, this value is adopted in the present study.

### 2.3 Theoretical maximum load-carrying capacity of RC beams

As given in Eq 15, the calculated maximum load-carrying capacity *Q*_*um*_ of the RC beam is obtained by summing the truss-action contribution *Q*_*t*_ from Eq 4 and the arch-action contribution *Q*_*a*_ from Eq 13.


$$\begin{array}{c} \;Q_{um}\text{\textasciitilde{}}=\;\xi a_{t}\sigma_{y}\frac{\text{1}}{\lambda_{d}}\text{+}\frac{\text{1}}{\text{8}}\beta_{a}\sigma_{B}b\frac{D}{\lambda_{D}} \end{array}$$ (15)

Similarly, the calculated maximum bending moment *M*_*um*_ corresponding to *Q*_*um*_ is obtained by summing the truss-action contribution *M*_*t*_ given by Eq 5 and the arch-action contribution *M*_*a*_ given by Eq 14, as expressed in Eq 16.


$$\begin{array}{c} \;M_{um}\text{\textasciitilde{}}=\;\xi a_{t}\sigma_{y}d+\frac{\text{1}}{\text{8}}\beta_{a}\sigma_{B}bD^{\text{2}} \end{array}$$ (16)

### 2.4 Comparison with code-based predictions

Fig 2 presents the schematic provided in the design code [31]and [33] for deriving the corresponding equations.

**Fig 2:**
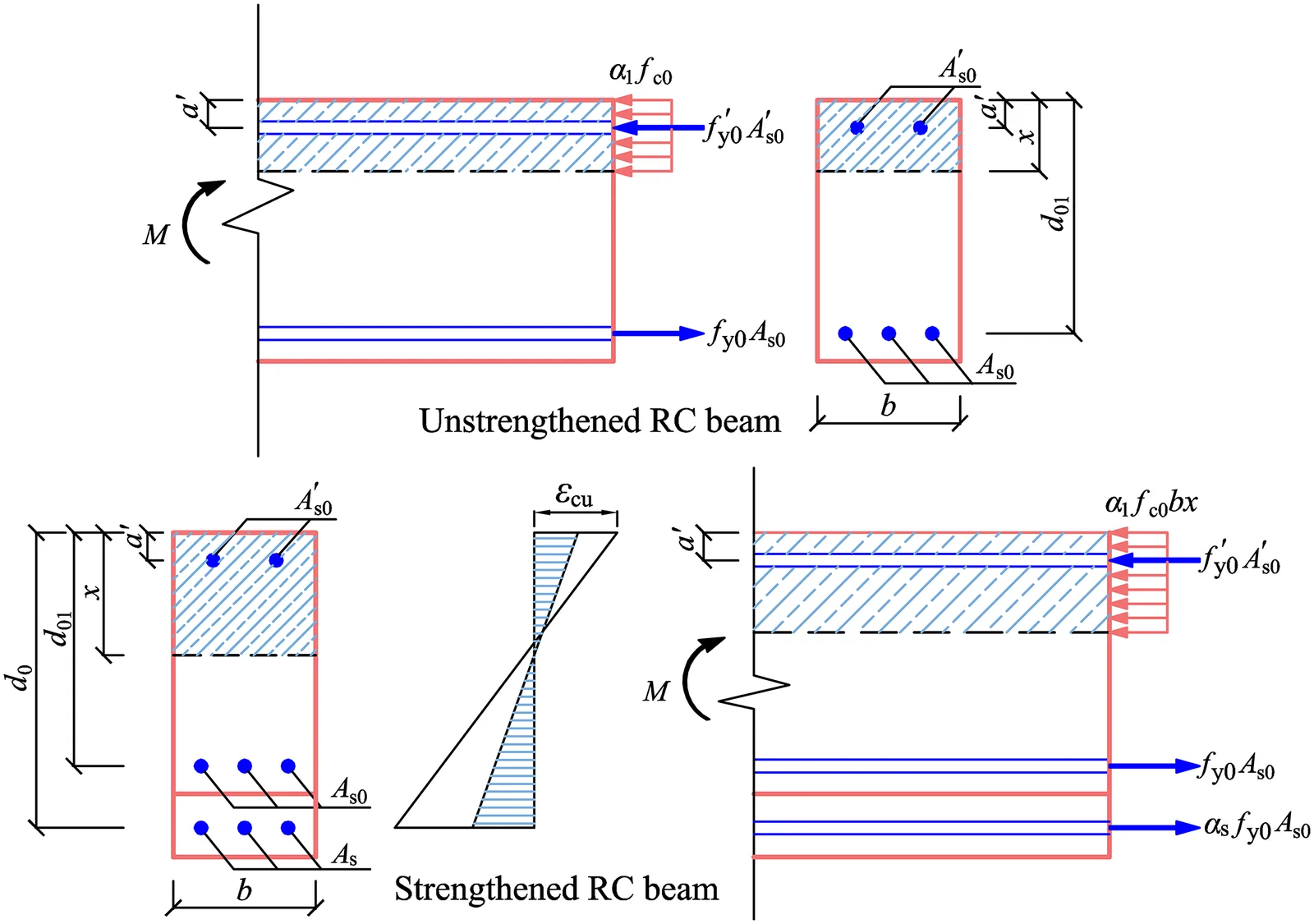
Schematic for the derivation of the code equations [31] and [33].

According to the *Code for Design of Concrete Structures* [33], the load-carrying capacity of unstrengthened RC beams can be calculated using the following equation:


$$\begin{array}{c} M⩽\alpha_{\text{1}}f_{\text{c0}}bx\left(d_{01}\text{-}\frac{x}{\text{2}}\right)\text{+}f_{\text{y0}}^{\prime }A_{\text{s0}}^{\prime }\left(d_{01}\text{-}a_{\text{1}}^{\prime }\right) \end{array}$$ (17)

$$\begin{array}{c} \alpha_{\text{1}}f_{\text{c0}}bx\text{\textasciitilde{}}=\;f_{\text{y0}}A_{\text{s0}}\text{-}f_{\text{y0}}^{\prime }A_{\text{s0}}^{\prime }\; \end{array}$$ (18)

$$\begin{array}{c} 2a^{\prime }⩽x⩽\xi_{\text{b}}d_{01} \end{array}$$ (19)

The applicable *Code for Design of Strengthening Concrete Structure* [31] specifies that the load-carrying capacity of rectangular-section flexural members strengthened in the tension zone can be calculated using the following equation:


$$\begin{array}{c} M\text{\textasciitilde{}}=\;\alpha_{\text{s}}f_{\text{y}}A_{\text{s}}\left(d_{\text{0}}\text{-}\frac{x}{\text{2}}\right)\text{+}f_{\text{y}0}A_{\text{s0}}\left(d_{01}\text{-}\frac{x}{\text{2}}\right)\text{+}f_{\text{y0}}^{\prime }A_{\text{s0}}^{\prime }\left(\frac{x}{\text{2}}\text{-}a^{\prime }\right) \end{array}$$ (20)

$$\begin{array}{c} \alpha_{\text{1}}f_{\text{c0}}bx\text{\textasciitilde{}}=\;f_{\text{y0}}A_{\text{s0}}\text{+}\alpha_{\text{s}}f_{\text{y}}A_{\text{s}}\text{-}f_{\text{y0}}^{\prime }A_{\text{s0}}^{\prime }\; \end{array}$$ (21)

$$\begin{array}{c} 2a^{\prime }⩽x⩽\xi_{\text{b}}d_{\text{0}} \end{array}$$ (22)

The parameter *a’* denotes the distance from the point of application of the resultant force in the longitudinal compression reinforcement to the extreme compression fiber of the concrete section. The parameter *α*_1_ is the equivalent rectangular stress-block coefficient for concrete in compression. It is taken as 1.0 for concrete strength classes not exceeding C50 and 0.94 for C80, with values for intermediate strength classes determined by linear interpolation.

Each strengthened specimen consists of an existing T-beam and a U-shaped RC jacket that together form a rectangular composite section. Because the longitudinal flexural reinforcement of each jacket is located in the tension zone, the strengthened beams fall within the scope of the aforementioned code provisions; their load-carrying capacities can therefore be calculated using the corresponding equations. The coefficient *ξ*_b_ in Eqs 19 and 22 is determined using Eq 23.


$$\begin{array}{c} \xi_{\text{b}}\text{\textasciitilde{}}=\;\frac{\beta_{\text{1}}}{\text{1+}\frac{\alpha_{\text{s}}f_{\text{sy}}}{\varepsilon_{\text{cu}}E_{\text{s}}}} \end{array}$$ (23)

In Eq 23, *β*_1_ is a code-specified coefficient. It is taken as 0.80 for concrete strength classes not exceeding C50 and 0.74 for C80; values for strength classes between C50 and C80 are determined by linear interpolation. The parameter *α*_s_ is the strength utilization coefficient of the tensile reinforcement and is taken as 1.0 for unstrengthened beams and 0.9 for strengthened beams. The parameter *ε*_cu_ denotes the ultimate compressive strain of concrete and is taken as *ε*_cu_ = 0.0033. The remaining symbols used in Eqs 17–23 are defined in Table 1.

**Table 1:** Definitions of symbols used in Eqs 17–23.

Symbol	Definition
*M*	Design bending moment of the beam
*α*_s_	Strength utilization coefficient of the tensile reinforcement
*f*_y_	Design tensile strength of the additional tensile reinforcement
*A*_s_	Cross-sectional area of the additional tensile reinforcement
*d*_0_	Effective depth of the strengthened beam section
*d*_01_	Effective depth of the original beam section
*x*	Depth of the concrete compression zone
*f*_y0_	Design tensile strength of the original tensile reinforcement
$f_{\text{y0}}^{\prime }$	Design compressive strength of the original compression reinforcement
*A*_s0_	Cross-sectional area of the original tensile reinforcement
$A_{\text{s0}}^{\prime }$	Cross-sectional area of the original compression reinforcement
*f*_c0_	Design compressive strength of the concrete in the original beam
*b*	Width of the rectangular section
*ξ*_b_	Relative depth of the limiting compression zone
*f*_sy_	Design tensile strength of the tensile reinforcement
*E*_s_	Elastic modulus of reinforcement

The code equations use the design strengths of concrete and steel reinforcement, which are lower than the corresponding characteristic strengths. Accordingly, the calculated flexural capacity is a design value and is generally conservative relative to the peak capacity measured experimentally. Although the underlying principle of the code method is straightforward, its application requires first determining the depth *x* of the concrete compression zone from sectional force equilibrium, checking *x* against the prescribed limits, and then selecting the applicable equation. The several parameters and conditional checks involved make the calculation procedure relatively cumbersome.

In contrast, although the derivation of the truss-arch model presented in this study requires the load-transfer mechanisms associated with the truss and arch actions to be established separately, the resulting expression is concise. Once the cross-sectional dimensions, material strengths, and shear-span ratio are specified, the maximum load-carrying capacity can be calculated directly without iteratively solving for the neutral-axis position. Thus, the model combines a clear mechanical basis with fewer calculation steps and facilitates parametric analyses and practical engineering applications.

## 3. Experimental program

### 3.1 Specimen design

The cyclic loading program comprised three RC beam specimens, designated BK, BG1, and BG2. Specimen BK was an unstrengthened T-beam designed to represent an existing beam in a building constructed around 2000. To reflect the material properties and reinforcement detailing typical of that period, BK was constructed using concrete with a compressive strength of 19.6 N/mm² and D6 and D16 reinforcing bars.

Specimens BG1 and BG2 were strengthened beams with rectangular composite sections formed by applying an 80-mm-thick U-shaped RC jacket to existing T-beams with the same original cross-section as specimen BK. The jacket concrete had a compressive strength of 34.8 N/mm². Four D20 reinforcing bars positioned at the corners of the jacket served as the main longitudinal reinforcement [31], while D6 bars were used as U-shaped stirrups. L-shaped anchor bars were anchored into both sides of the existing beams and welded to the U-shaped stirrups using 70-mm-long welds [31], as shown in Fig 3.

**Fig 3:**
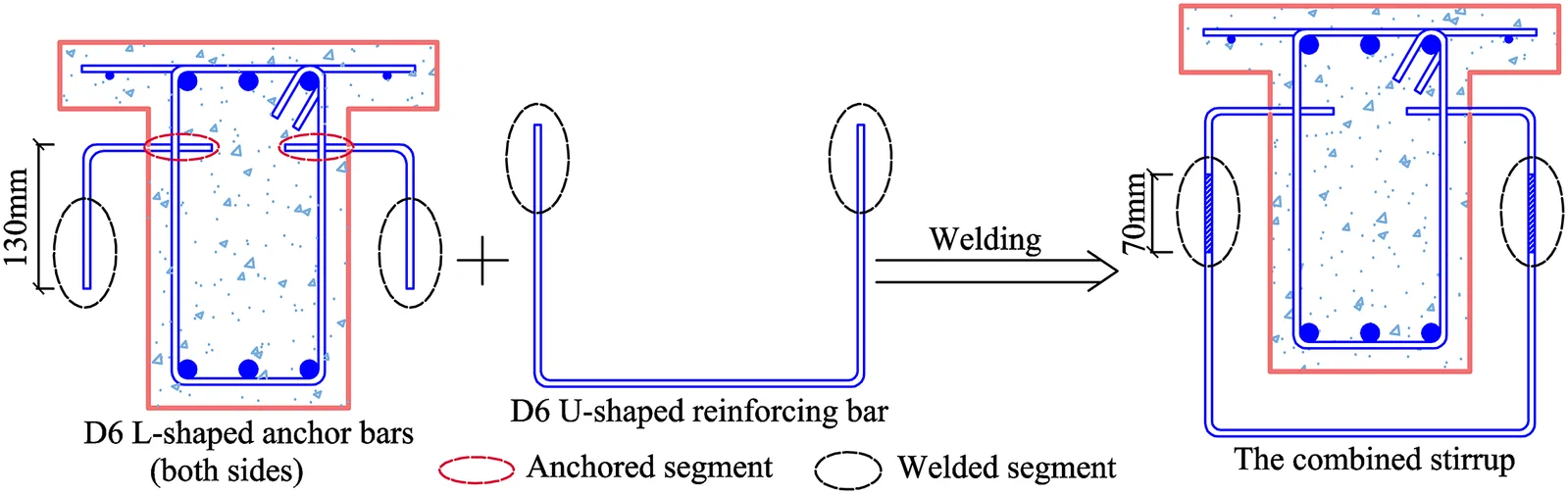
Schematic of stirrup detailing in the U-shaped RC jacket.

The key difference between specimens BG1 and BG2 was the treatment of the existing concrete surface before jacketing. The interface of BG1 was roughened, whereas that of BG2 was left unroughened. This comparison was intended to provide a preliminary assessment of the influence of interface roughness on strengthening effectiveness rather than a comprehensive investigation of interface behavior. The cross-sectional details of the three specimens are shown in Fig 4.

**Fig 4:**
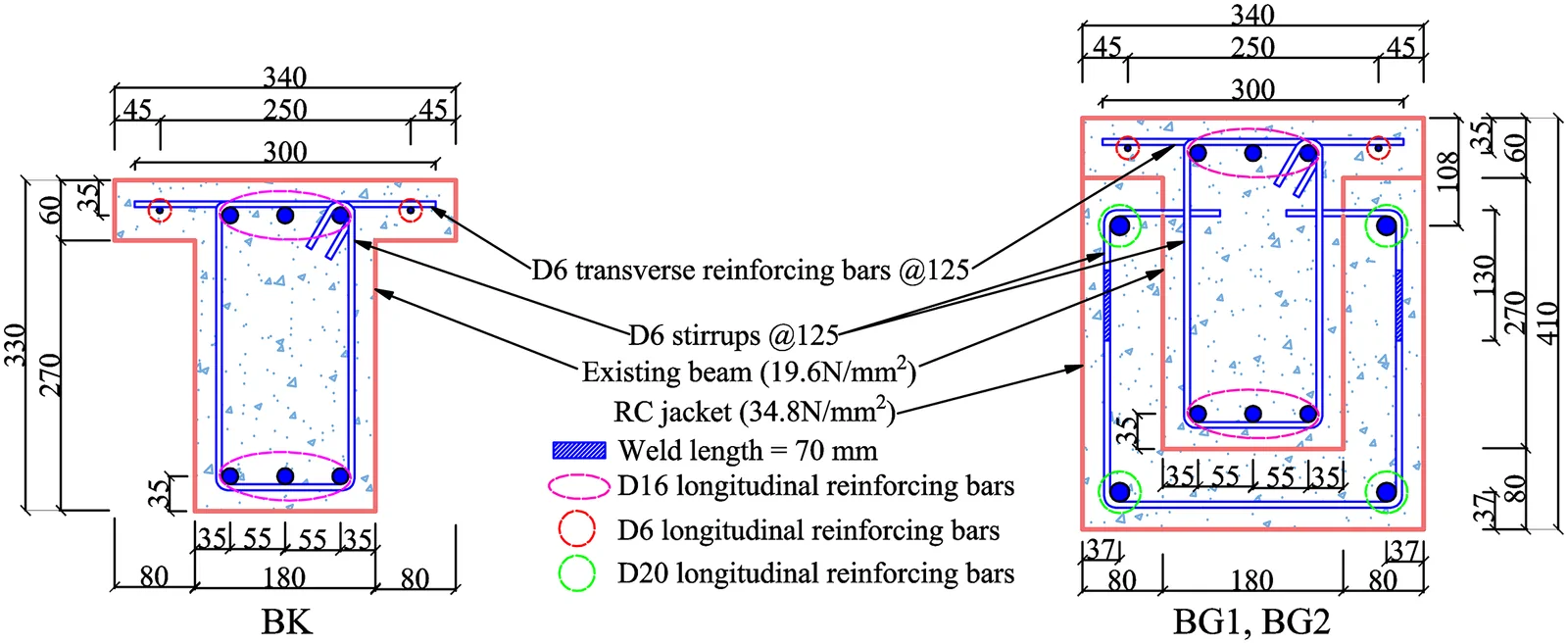
Cross-sectional details of specimens BK, BG1, and BG2 (dimensions in mm).

In this study, the overall strengthening effectiveness of the U-shaped RC jacket was evaluated by comparing BK with BG1 and BG2. The influence of interface roughening on the composite action between the existing and jacket concrete was assessed by comparing BG1 with BG2. Similar experimental schemes comprising one control beam and two strengthened beams have been adopted in exploratory studies of RC beam strengthening [4,34]. Study [35] likewise employed three-specimen comparative series for different cross-sectional configurations. Accordingly, the three-specimen test program is comparable in scale to similar studies of strengthening mechanisms and is adequate for a preliminary assessment of strengthening feasibility and key load-resisting characteristics. However, the limited sample size does not permit a statistical evaluation of experimental variability or comprehensive validation of the broader applicability of this strengthening method.

### 3.2 Material properties

Three reinforcing-bar sizes were used in the specimens: D6, D16, and D20, where D denotes diameter and the numeral indicates the diameter in millimeters. Tensile tests were conducted on each bar size in accordance with the relevant standard [36]. The measured yield strengths, ultimate tensile strengths, and elongations, together with the calculated yield strains, are summarized in Table 2. The yield strain was calculated by dividing the yield strength by the elastic modulus of steel, which was taken as 205000 N/mm².

**Table 2:** Mechanical properties of reinforcing bars.

Type	Diameter (mm)	Yield strength (N/mm²)	Yield strain (%)	Ultimate Strength (N/mm²)	Elongation (%)
D6	6	342	0.17	445	20
D16	16	333	0.16	464	24
D20	20	391	0.19	547	18

To characterize the mechanical properties of the existing-beam concrete and the jacket concrete, tests were conducted in accordance with GB/T 50081−2019 [37]. Cylindrical concrete specimens, 150 mm in diameter and 300 mm in height, were cured for 28 days at 20 ± 5 °C and 95% relative humidity and then tested to determine their compressive and tensile strengths. The mix proportions of the existing-beam and jacket concrete mixtures are listed in Table 3, and the corresponding compressive and tensile strength test results are presented in Table 4.

**Table 3:** Concrete mix proportions.

Type	Cement (kg/m^3^)	Water (kg/m^3^)	Fine aggregate (kg/m^3^)	Coarse aggregate (kg/m^3^)	water-reducing admixture (kg/m^3^)
Existing beam	250	200	885	861	2.5
Jacket concrete	383	180	812	879	2.99

**Table 4:** Compressive and tensile strengths of concrete.

Type	Compressive strength (N/mm²)	Compressive strain (%)	Tensile strength (N/mm²)	Elastic modulus (N/mm²)
Existing beam	19.6	0.17	2.1	24600
Jacket concrete	34.8	0.23	2.9	29400

### 3.3 Specimen preparation

The beams were fabricated and cured in accordance with GB 50666-2011 [38]. Following casting, they were moist-cured for 28 days. While the formwork remained in place, the formwork and its exposed surfaces were kept moist. Immediately after formwork removal, the beam flanges, both faces of the webs, and the soffits were covered with wet cloths and plastic sheeting. Water was sprayed as needed to prevent the coverings from drying and to keep the concrete surfaces continuously moist. The curing temperature was maintained at 20 ± 5 °C.

For BG1, the existing concrete surfaces within the area to be covered by the U-shaped RC jacket were mechanically roughened using a chisel and pneumatic hammer. The surface cement paste and loose material were removed to expose the coarse aggregate, resulting in a roughening depth of approximately 8 mm. In contrast, the corresponding concrete surfaces of BG2 were retained in their original condition without roughening.

Following surface preparation, anchor holes 8 mm in diameter and 60 mm deep were drilled at the designated locations on both sides of the two beams to be strengthened. The holes were cleaned using a compressed-air gun and a brush, after which D6 L-shaped anchor bars were bonded into the holes using structural anchoring adhesive. D6 U-shaped stirrups were arranged along the beam at 125 mm spacing and connected to the L-shaped anchor bars using 70-mm-long single-sided welds, thereby forming the stirrup system of the U-shaped RC jacket. Subsequently, one D20 longitudinal reinforcing bar was placed at each of the four corners of the jacket, followed by completion of the reinforcement cage and installation of the formwork.

The interface surfaces of both beams to be strengthened were cleaned with wire brushes to remove dust, debris, and loose particles, after which a bonding agent was applied. Finally, jacket concrete was cast into the formwork and cured for 28 days.

### 3.4 Cyclic loading and deflection measurement

The loading experiments were conducted in accordance with GB/T 50152−2012 [39]. The specimens were tested as simply supported beams under symmetric two-point concentrated loading. The two loading points were spaced 1000 mm apart, and the distance between the supports was 2500 mm. The deflection-controlled loading protocol is shown in Fig 5(a). The control deflection was defined as the difference between the vertical deflection at midspan and the mean vertical displacement of the two supports. Cyclic loading was applied sequentially at deflection amplitudes of ±2, ± 4, ± 8, ± 16, and ±32 mm, followed by a final positive loading stage to +64 mm.

**Fig 5:**
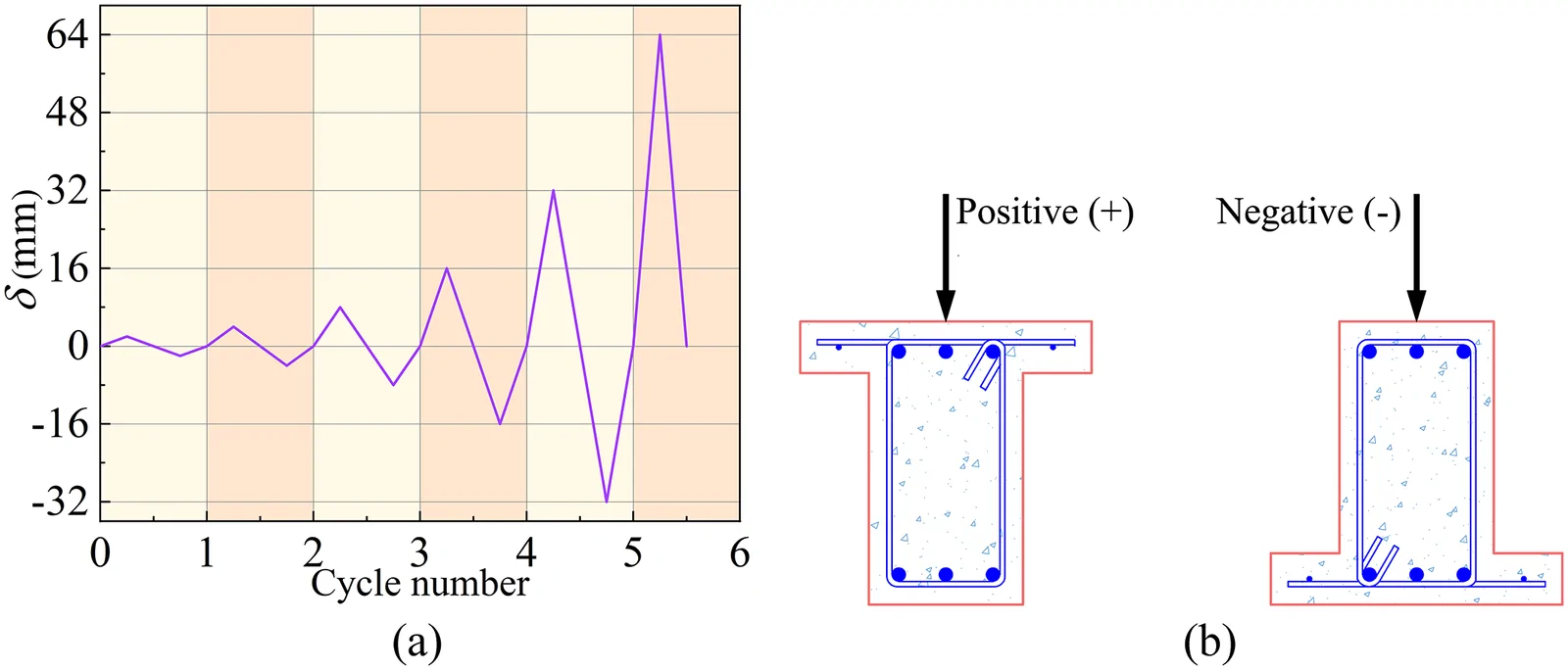
Loading protocol. (a) Deflection amplitude. (b) positive and negative loading configurations.

Deflection-controlled loading was applied in stages as follows: (1) for target deflections of ±2 and ±4 mm, loading proceeded in 1 mm increments at a rate of 2 mm/min, and each successive deflection level was maintained for 5 min before proceeding to the next increment; (2) for target deflections of ±8 and ±16 mm, loading proceeded in 4 mm increments at a rate of 4 mm/min, and each successive deflection level was maintained for 8 min; and (3) for target deflections of ±32 and +64 mm, loading proceeded in 8 mm increments at a rate of 8 mm/min, and each successive deflection level was maintained for 10 min.

Positive and negative loading were achieved by inverting the specimens. Positive loading was defined as the configuration in which the flange of the original T-beam was in compression, whereas negative loading corresponded to the configuration in which the flange was in tension. The two loading configurations are shown in Fig 5(b).

The experimental setup is shown in Fig 6(a) and 6(b). In Fig 6(a), labels 1 ~ 6 identify the deflection gauges. Gauges 1 ~ 5 were mounted along a horizontal line at the mid-depth of the beam and positioned at the two supports, the two loading points, and midspan. Gauge 6 was installed on the beam soffit at midspan.

**Fig 6:**
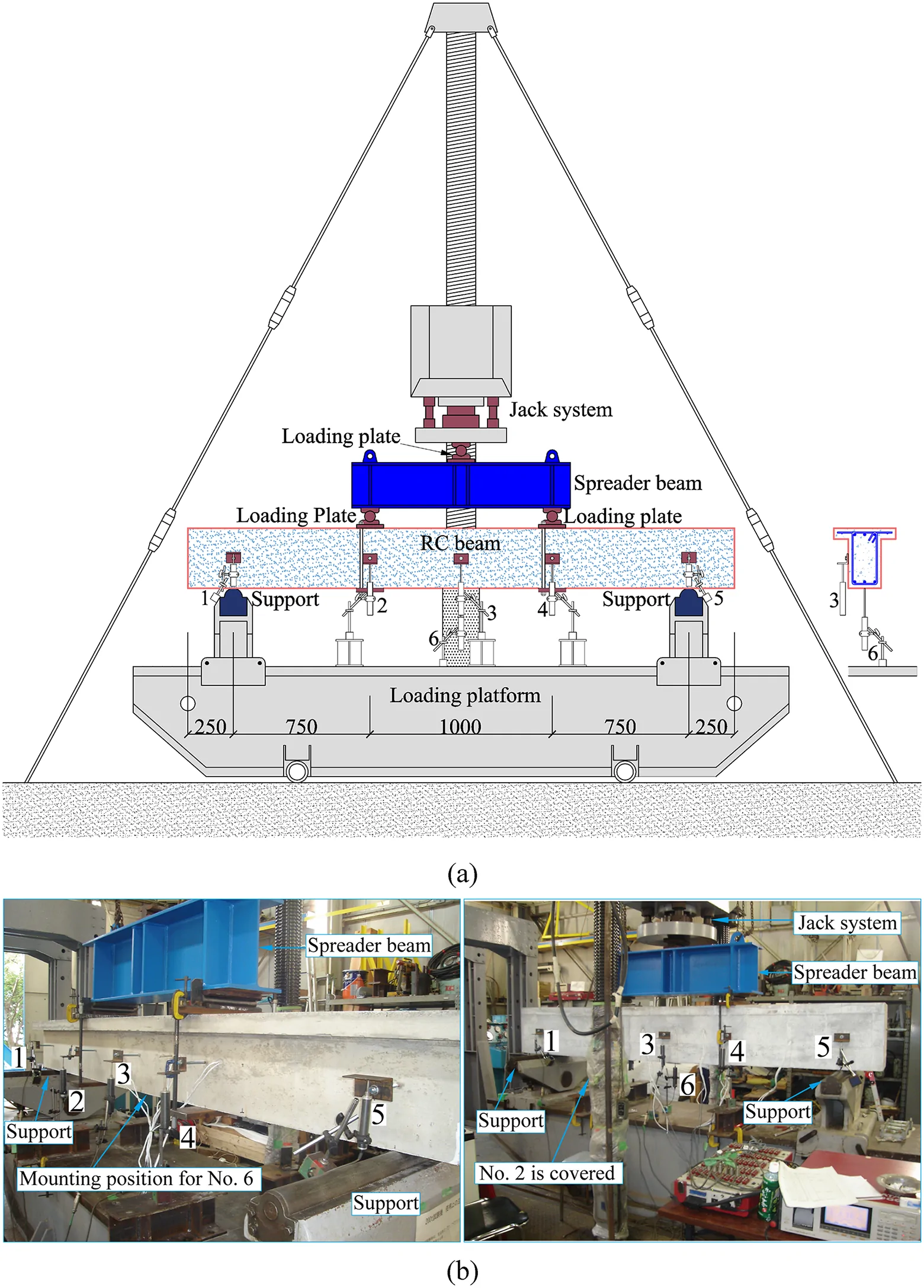
Loading setup. (a) Schematic of the experimental setup (dimensions in mm). (b) Photograph of the experimental setup.

### 3.5 Correction for deflection measurement

In this study, a loading round was defined as loading from zero load to the prescribed maximum deflection in one direction, followed by complete unloading to zero load. For each loading round, the deflection at each measurement point was calculated from the readings of all deflection gauges, with corrections applied to account for residual deflection from the preceding round and variations in support displacements.

#### 3.5.1 Correction for residual deflection from the preceding loading round.

As shown in Fig 7, the uncorrected residual deflections at the end of successive loading rounds are denoted by *w*_1_, *w*_2_, *w*_3_, …, *w*_k_, where *w* denotes the deflection at an arbitrary point and k is a positive integer satisfying 1 ≤ k ≤ 11. The corresponding residual deflections after correction are denoted by *x*_1_, *x*_2_, *x*_3_, …, *x*_k_, while the corrected deflections at arbitrary points within the respective loading rounds are denoted by *y*_1_, *y*_2_, *y*_3_, …, *y*_k_. For k = 1, no preceding loading round exists; therefore, no residual-deformation correction is required, and *y*_1_ = *w*_1_. The relationships between the uncorrected and corrected residual deflections for k > 2 are presented in Table 5.

**Table 5:** Deflection-correction relationship.

The value of k	Before correction	After correction
2, 4, 6, 8, 10	*w*_k_=*w*_k-1_-Δ*w*_k_	*y*_k_=*x*_k-1_-(*w*-*w*_k-1_)
3, 5, 7, 9, 11	*w*_k_=*w*_k-1_+Δ*w*_k_	*y*_k_=*x*_k-1_+(*w*-*w*_k-1_)

**Fig 7:**
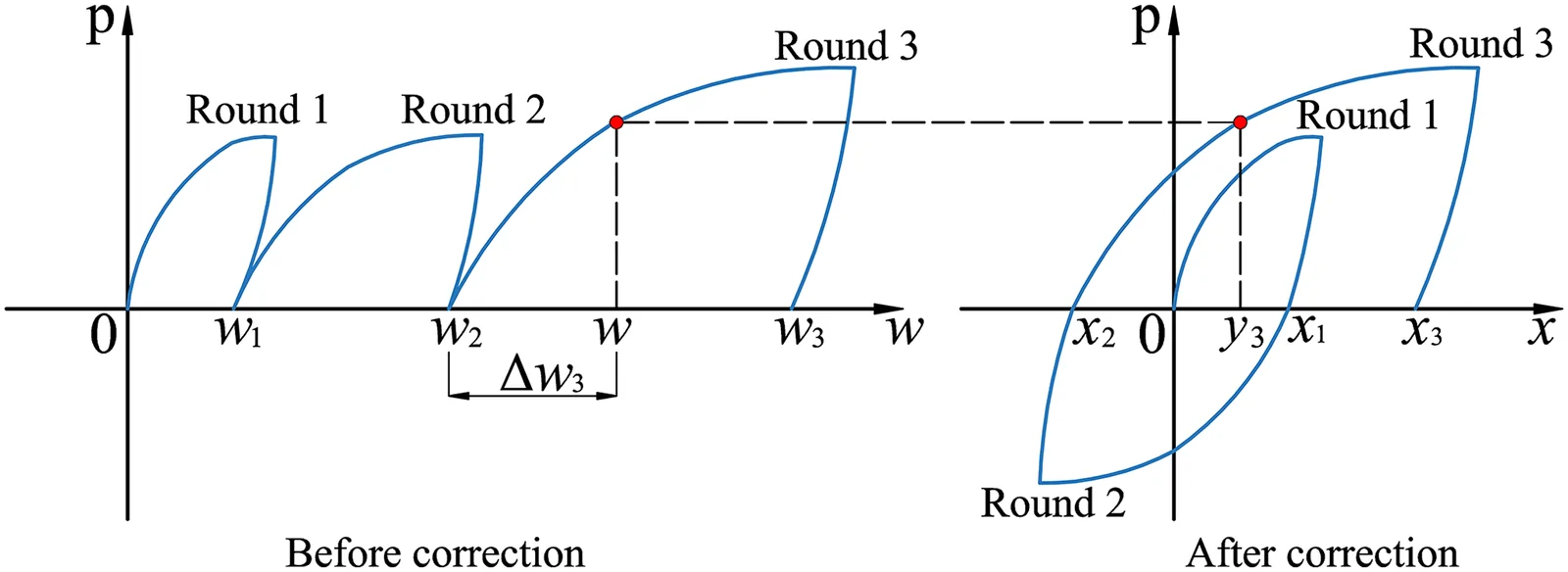
Correction for residual deflection.

#### 3.5.2 Correction for variations in support displacements.

As shown in Fig 8, the two supports, two loading points, and midspan are designated as points a ~ e, respectively, and their deflections are denoted by *y*_a_ ~ *y*_e_. Here, *y* has the same meaning as *y*_k_ in Section 3.5.1. The support-displacement correction values at these points are denoted by *m*_a_ ~ *m*_e_. Table 6 gives the relationships between *m*_a_ ~ *m*_e_ and *y*_a_ ~ *y*_e_, while Table 7 presents the corresponding deflections *δ*_a_ ~ *δ*_e_ relative to the deflections at the two support points.

**Table 6:** Deflection-correction values at each point.

Correction of *y*_a_ and *y*_e_	The correction value for *m*_a_-*m*_e_				
	*m*_a_	*m*_b_	*m*_c_	*m*_d_	*m*_e_
*y*_a_→0	*y*_a_	0.7*y*_a_	0.5*y*_a_	0.3*y*_a_	0
*y*_e_→0	0	0.3 *y*_e_	0.5*y*_e_	0.7*y*_e_	*y*_e_
*y*_a_, *y*_e_→0	*y*_a_	0.7*y*_a_ + 0.3*y*_e_	0.5*y*_a_ + 0.5*y*_e_	0.3*y*_a_ + 0.7*y*_e_	*y*_e_

**Table 7:** Deflections at points a ~ e relative to the supports.

Relative deflection	*δ*_a_	*δ*_b_	*δ*_c_	*δ*_d_	*δ*_e_
Values	0	*y*_b_-0.7*y*_a_-0.3y_e_	*y*_c_-0.5*y*_a_-0.5*y*_e_	*y*_d_-0.3*y*_a_-0.7y_e_	0

**Fig 8:**
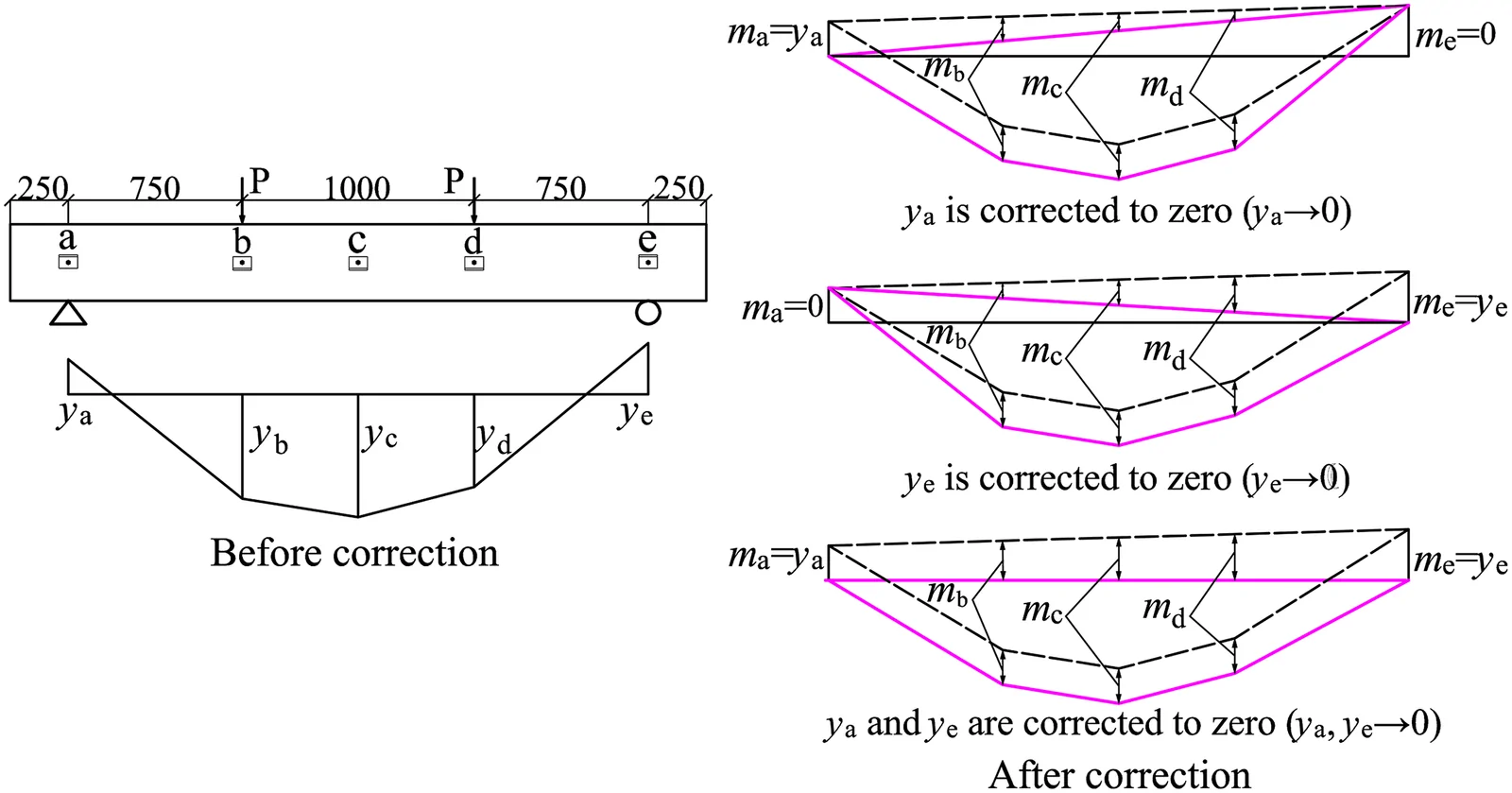
Support-displacement correction (all dimensions are in mm).

## 4. Results and discussion

### 4.1 Results of theoretical calculations

Table 8 compares the maximum experimental loads with the results obtained using the proposed truss-arch model and the code-based method, while Table 9 presents the respective contributions of the truss and arch actions to *Q*_*um*_.

**Table 8:** Comparison of calculated load-carrying capacities.

Specimen		Experimental value (kN)	Truss–arch (*Q*_*um*_)		Code-based value (2*M*/*l*_*s*_)	
			value (kN)	Relative error (%)	value (kN)	Relative error (%)
BG1	+	465.52	464.20	0.28	348.58	25.12
	–	−452.90	−485.98	7.30	--	--
BG2	+	472.42	464.20	1.74	348.58	26.21
	–	−450.31	−485.98	7.92	--	--
BK	+	177.43	160.12	9.76	125.46	29.29
	–	−181.63	−176.80	2.66	--	--

**Table 9:** Calculated values of *Q*_*t*_ and *Q*_*a*_.

Specimen		*Q*_*um*_ = *Q*_*t*_ + *Q*_*a*_ (kN)	Truss action *Q*_*t*_		Arch action *Q*_*a*_	
			Values (kN)	Proportion (%)	Values (kN)	Proportion (%)
BG1, BG2	+	464.20	386.50	83	77.70	17
	–	−485.98	−408.28	84	−77.70	16
BK	+	160.12	140.88	88	19.24	12
	–	−176.80	−157.56	89	−19.24	11

As shown in Table 8, the positive-direction load values calculated using the code-based method were 348.58 kN for both strengthened beams and 125.46 kN for the unstrengthened beam. These values were 25.12%, 26.21%, and 29.29% lower than the experimental maximum loads of BG1, BG2, and BK, respectively. The proposed truss-arch model predicted corresponding capacities of 464.20 kN for the strengthened beams and 160.12 kN for the unstrengthened beam, with relative errors of 0.28%, 1.74%, and 9.76%, respectively. The code-based method uses design values of material strengths and incorporates safety margins because it is intended primarily for engineering design, whereas the proposed model uses measured material properties to predict the maximum loads of the test specimens.

For BG1 and BG2, the relative errors under positive loading were small, at 0.28% and 1.74%, respectively. Under negative loading, the theoretical maximum load was −485.98 kN, whereas the maximum experimental loads were −452.90 kN and −450.31 kN, corresponding to relative errors of 7.30% and 7.92%, respectively. Thus, the negative-loading results exhibited larger discrepancies than the positive-loading results. This difference was primarily attributable to the loading protocol: the maximum negative deflection was limited to −32 mm, only half of the + 64 mm imposed under positive loading. Consequently, neither specimen had reached its actual maximum negative load when loading was terminated at −32 mm.

For BK, the theoretical maximum loads under positive and negative loading were 160.12 kN and −176.80 kN, respectively, whereas the corresponding maximum experimental loads were 177.43 kN and −181.63 kN. The resulting relative errors were 9.76% and 2.66%, respectively. Compared with BG1 and BG2, BK exhibited a larger error under positive loading because the theoretical model idealizes its T-shaped section as a rectangular section with a width of 180 mm and a height of 330 mm. This idealization excludes part of the flange concrete from the calculation, resulting in a smaller calculated concrete area and a conservative prediction.

As shown in Table 9, the truss action contributes more than 80% of the maximum load-carrying capacity predicted by the model, substantially exceeding the contribution of the arch action. Therefore, for the beams examined in this study, the truss action is the dominant load-resisting mechanism and plays the primary role in determining the maximum load-carrying capacity.

These results demonstrate only that the proposed model provides good predictive accuracy for the specimens and loading conditions examined in this study; they do not establish that the model can replace the code provisions. Further validation against independent experimental data is required for members involving different parameter combinations, severe pre-existing damage, interface debonding, asymmetric loading, or brittle shear failure. Until a larger independent experimental database is available and appropriate reliability factors have been calibrated, the proposed model should be used as a supplement to, rather than a substitute for code-based calculations.

### 4.2 Comparison of key loads

Table 10 summarizes the experimental results, including the loads and midspan deflections corresponding to the onset of flexural and shear cracking in each specimen. In the table, + 1 and −1 denote the positive and negative loading directions, respectively, within the first loading cycle. Subsequent loading cycles follow the same notation. The symbol *δ* denotes the corresponding midspan deflection.

**Table 10:** Summary of experimental results.

Observed event		BG1			BG2			BK		
		*P* (kN)	*δ* (mm)	Cycle	*P* (kN)	*δ* (mm)	Cycle	*P* (kN)	*δ* (mm)	Cycle
Flexural crack	+	49.47	0.26	+1	103.86	0.60	+1	43.86	0.86	+1
	–	−98.63	−0.40	−1	−103.57	−0.40	−1	−51.33	−0.64	−1
Shear crack	+	251.55	3.89	+2	167.85	1.70	+1	116.66	4.02	+2
	–	−263.17	−2.92	−2	−202.47	−2.02	−1	−116.62	−3.52	−2

As shown in Table 10, BG1 and BG2 exhibited similar flexural-cracking loads at all loading stages except +1. Nevertheless, the flexural-cracking loads of both strengthened specimens were higher than those of BK at every stage. The flexural-cracking load of BG1 at +1 was only 49.47 kN, possibly because of local defects in the specimen. The shear cracking loads of BG1 were approximately 49.9% and 30.0% higher than those of BG2 under positive and negative loading, respectively. This difference may be attributed to surface roughening, which enhanced bonding and mechanical interlock between the existing and jacket concrete, thereby improving composite action and shear cracking resistance. Table 11 presents the loads recorded at the maximum control deflection in each loading direction of every loading cycle.

**Table 11:** Loads at the maximum control deflection (kN).

Specimen	Cycle 1		Cycle 2		Cycle 3		Cycle 4		Cycle 5		Cycle 6
	+1	−1	+2	−2	+3	−3	+4	−4	+5	−5	+6
BG1	148	−155	252	−318	379	−417	411	−421	432	−453	466
BG2	192	−202	299	−334	388	−426	421	−433	435	−450	472
BK	67	−84	117	−127	171	−180	171	−180	166	−182	177
BG2/BG1	1.30	1.30	1.19	1.05	1.02	1.02	1.02	1.03	1.01	0.99	1.01
BG1/BK	2.21	1.85	2.15	2.50	2.22	2.32	2.40	2.34	2.60	2.49	2.63
BG2/BK	2.87	2.40	2.56	2.63	2.27	2.37	2.46	2.41	2.62	2.47	2.67

As shown in Table 11, the BG2-to-BG1 load ratio at the maximum control deflection was relatively high during the first two loading cycles, reaching a maximum of 1.30. As the control-deflection amplitude increased, this ratio gradually decreased to 1.01. All BG1-to-BK and BG2-to-BK load ratios exceeded 2.0, except for the BG1-to-BK ratio at the −1 loading stage. These results indicate that strengthening with a U-shaped RC jacket effectively improved the load-carrying performance of the tested beams. However, comparison of BG1 and BG2 alone is insufficient to determine the effect of interface roughening on the load-carrying capacity of strengthened beams; further verification using a larger number of replicate specimens is required.

### 4.3 Analysis of hysteretic curves

The hysteretic curves of the three specimens are shown in Fig 9. All three specimens exhibited full hysteresis loops without pronounced pinching. Even after the absolute midspan deflection exceeded 30 mm, the load magnitude continued to increase with increasing deflection amplitude. The specimens therefore retained substantial load-carrying capacity at large deformations, with no abrupt strength loss characteristic of brittle failure, indicating ductile flexural behavior.

**Fig 9:**
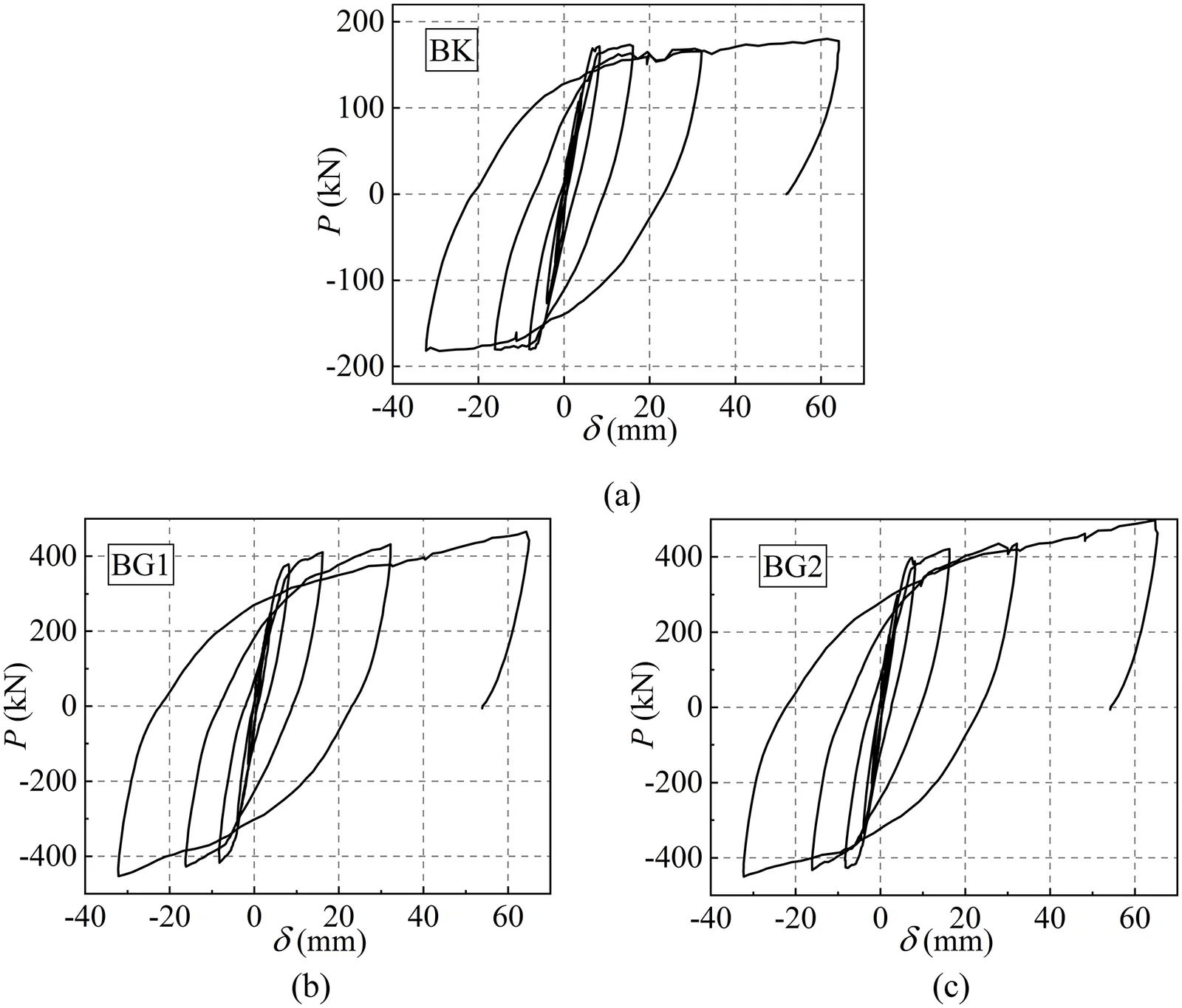
Experimental load–deflection hysteretic curves of the three specimens. (a) BK. (b) BG1. (c) BG2.

The following sections further evaluate the structural performance of the specimens in terms of stiffness, ductility coefficient, and cumulative energy dissipation and examine their failure modes.

#### 4.3.1 Stiffness analysis.

According to reference [40], the stiffness of a specimen can be characterized by its secant stiffness. The secant stiffness and its degradation curve reflect the evolution of damage during cyclic loading. A slower rate of stiffness degradation generally indicates more gradual and distributed damage development, resulting in a more stable structural response. The secant stiffness and stiffness degradation coefficient are calculated using Eqs 24 and 25, respectively.


$$\begin{array}{c} K_{i}=\frac{\left|+F_{i}|+|-F_{i}\right|}{\left|+X_{i}|+|-X_{i}\right|} \end{array}$$ (24)

$$\begin{array}{c} D_{i}=\frac{K_{\text{0}}-K_{i}}{K_{\text{0}}} \end{array}$$ (25)

In Eq 24, *K*_*i*_ represents the secant stiffness (kN/mm) in the *i* th loading cycle; + *F*_*i*_ and −*F*_*i*_ denote the positive and negative peak loads, respectively, in that cycle; and +*X*_*i*_ and −*X*_*i*_ denote the corresponding midspan deflections. In Eq 25, *D*_*i*_ is the stiffness degradation coefficient, while *K*_0_ is the initial stiffness, defined as the secant stiffness in the first loading cycle. A larger *D*_*i*_ indicates greater stiffness degradation.

Because cyclic loading was controlled by the midspan deflection, a deflection-based method was used to decompose the hysteretic curves. Each complete loading cycle comprised the specimen being loaded from zero load to the prescribed maximum positive deflection and then unloaded to zero load, followed by loading to the corresponding maximum negative deflection and unloading again to zero load. Together, these stages formed one complete hysteresis loop corresponding to one loading cycle in Fig 5(a). The secant stiffness values and stiffness degradation coefficients of the three specimens are summarized in Table 12 and plotted in Fig 10.

**Table 12:** Secant stiffness and stiffness degradation coefficients of the specimens.

Loop	BG1		BG2		BK	
	*K*_*i*_ (kN/mm)	*D*_*i*_	*K*_*i*_ (kN/mm)	*D*_*i*_	*K*_*i*_ (kN/mm)	*D*_*i*_
1	86.47	0.00	93.55	0.00	36.86	0.00
2	71.19	0.18	78.25	0.16	30.24	0.18
3	47.94	0.45	49.87	0.47	21.37	0.42
4	25.84	0.70	26.32	0.72	10.97	0.70
5	13.74	0.84	13.73	0.85	5.45	0.85
6	7.17	0.92	7.61	0.92	2.81	0.92

**Fig 10:**
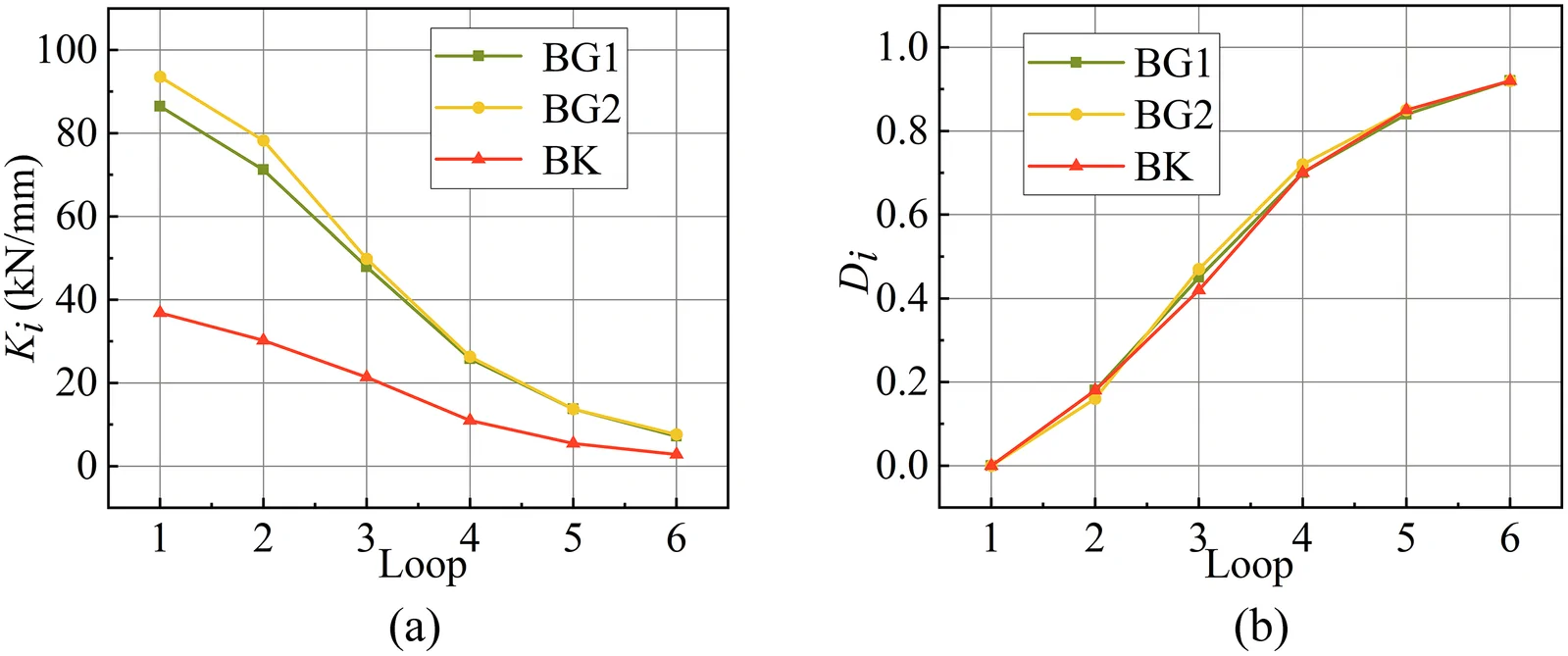
Stiffness variations. (a) Secant stiffness. (b) Stiffness degradation coefficient.

As shown in Table 12 and Fig 10, the secant stiffness of all three specimens decreased progressively with successive loading stages. The reduction was rapid during the early stages as cracking developed, whereas the degradation curves gradually flattened during the later stages because the specimens had already accumulated substantial damage and retained relatively low stiffness. This trend reflects the irreversible accumulation of damage in RC beams under cyclic loading, with each loading cycle producing further stiffness loss.

The strengthened specimens BG1 and BG2 exhibited substantially higher stiffness than the unstrengthened specimen BK. Across the six reported loading stages, their secant stiffnesses remained 2.2–2.7 times the corresponding stiffness of BK, demonstrating that the strengthening scheme increased stiffness from the initial loading stage onward and maintained this advantage throughout loading. The final stiffness degradation coefficient was 0.92 for all three specimens, indicating severe stiffness degradation consistent with a near-failure state.

#### 4.3.2 Yield points and ductility coefficients.

The deflection ductility coefficient (*μ*) is a dimensionless performance index defined as the ratio of the ultimate deflection to the yield deflection. It quantifies the ability of a beam to undergo plastic deformation without a marked post-yield loss of load-carrying capacity and is widely used to assess seismic performance. According to Reference [40], *μ* is calculated using Eq 26.


$$\begin{array}{c} \mu =\frac{\delta_{\text{u}}}{\delta_{\text{y}}} \end{array}$$ (26)

In Eq 26, *μ* denotes the deflection ductility coefficient; *δ*_u_ is the ultimate deflection, defined as the deflection on the descending branch at which the load has decreased to 85% of the peak load; and *δ*_y_ is the yield deflection corresponding to the yield load. For an RC beam, the yield point is defined as the point at which the member develops global yielding, accompanied by a marked reduction in stiffness.

The Park method [41] was used to determine the yield point as illustrated in Fig 11. First, the peak load was identified at point D, and point A was located on the ascending branch at 75% of the peak load. A straight line was then drawn from the origin O through point A and extended to intersect the horizontal line passing through point D at point C. A vertical line through point C intersected the load–deflection curve at point B, which was defined as the yield point; the corresponding abscissa was the yield deflection *δ*_y_. Point E was defined as the ultimate point, at which the load on the descending branch had decreased to 85% of the peak load; the corresponding abscissa was the ultimate deflection *δ*_u_

**Fig 11:**
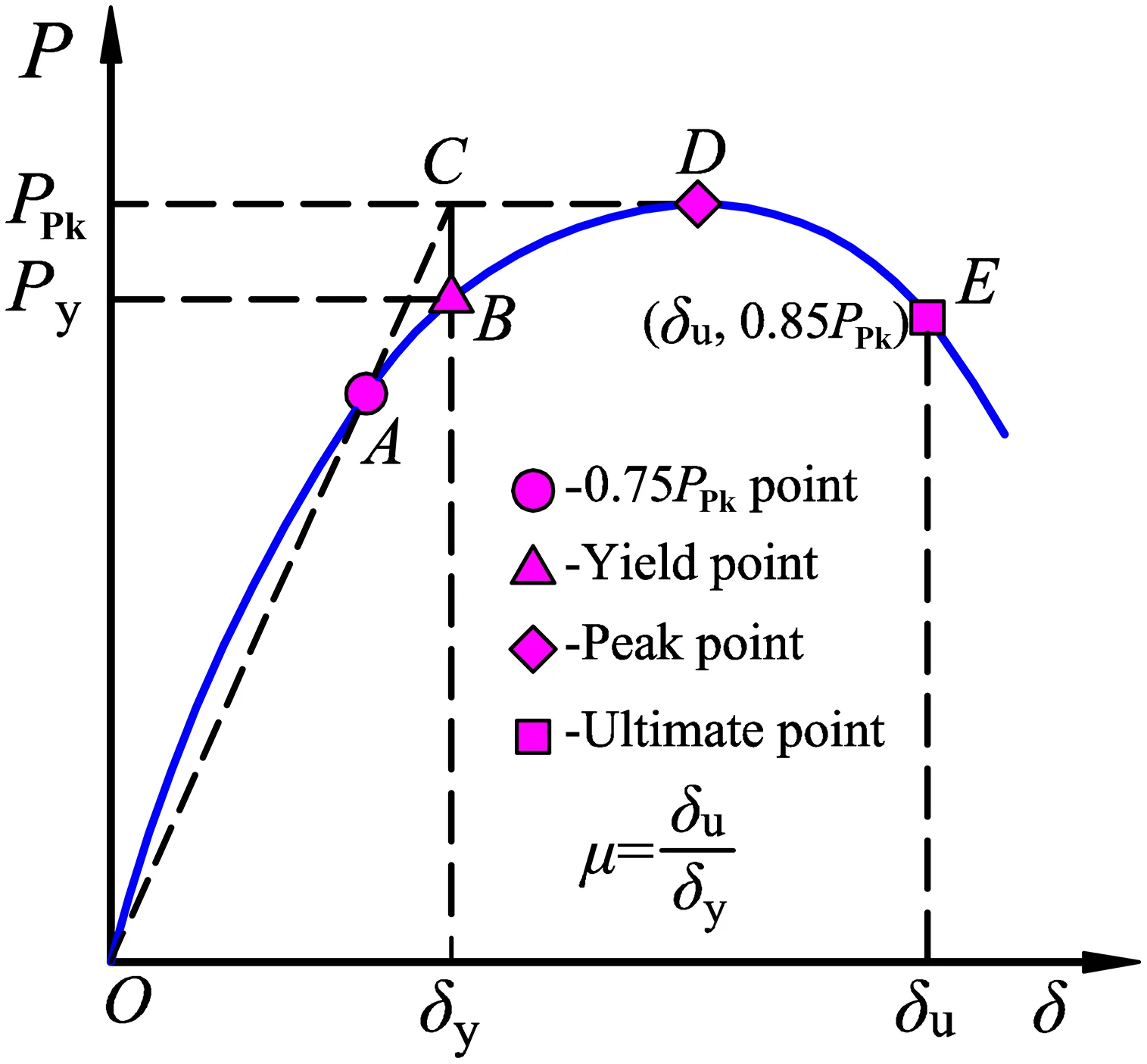
Determination of the yield point and calculation of the ductility coefficient.

Because the maximum negative control deflection was limited to −32 mm, which was insufficient for the specimens to reach their ultimate deflections, only the positive-direction ductility coefficients were evaluated. Moreover, none of the specimens reached the post-peak load-drop criterion of 0.85*P*_Pk_ before completion of the loading program. Therefore, for the purpose of calculating the ductility coefficients, the final stabilized load and deflection recorded at the + 64 mm loading stage were adopted as the nominal ultimate load and ultimate deflection, respectively. Table 13 presents the calculated ductility coefficients.

**Table 13:** Calculated deflection ductility coefficients.

Specimen	Yield deflection (mm)	Yield load (kN)	Ultimate deflection (mm)	Ultimate load (kN)	Ductility coefficient
BG1	8.00	378.08	64.96	442.25	8.12
BG2	8.13	399.76	65.33	463.23	8.04
BK	6.43	165.85	64.13	167.67	9.98

As shown in Table 13, the positive-direction deflection ductility coefficients of all three specimens exceeded 3. According to reference [42], beams with ductility coefficients greater than 3 exhibit good ductility and can satisfy seismic performance requirements. Therefore, all three specimens demonstrated favorable positive-direction ductility. Like the unstrengthened specimen BK, the strengthened specimens BG1 and BG2 exhibited ductile failure, indicating that the U-shaped RC jacket did not alter the failure mode. Overall, the strengthening scheme effectively increased stiffness while preserving the ductile behavior of the beams.

#### 4.3.3 Cumulative energy dissipation.

The energy dissipation capacity of a beam refers to its ability to absorb and dissipate input energy through plastic deformation, frictional sliding, and crack propagation, opening, and closure under seismic or cyclic loading. It is an important indicator of the seismic performance of a structural member. At the *i* th control-deflection level, the dissipated energy *E*_*i*_ is defined as the area enclosed by the complete loading–unloading path in the load-deflection plane. For numerical evaluation, the experimental data at each control-deflection level were arranged in their recording sequence, and the enclosed area was calculated using trapezoidal integration:


$$\begin{array}{c} E_{i}\text{\textasciitilde{}}=\;\left|\sum_{j=1}^{m_{i}}\frac{P_{i,j}\text{+}P_{i,j+1}}{\text{2}}\left(\delta_{i,j+1}\text{-}\delta_{i,j}\right)\right| \end{array}$$ (27)

In the equation, *P*_*i*, *j*_ and *δ*_*i*, *j*_ denote the load and midspan deflection, respectively, at the *j* th data point of the *i* th control-deflection level; *m*_*i*_ is the number of data points forming the corresponding hysteresis loop. To close the loop numerically, the *m*_*i*_ + 1 th point is set equal to the first point. The absolute value of the entire summation is taken to eliminate the effect of loading direction on the sign of the dissipated energy. The cumulative energy dissipation *E*_*cum*, *n*_ after completion of the first *n* control-deflection levels is calculated as follows:


$$\begin{array}{c} E_{\text{cum},\;n}\text{\textasciitilde{}}=\;\sum_{i=1}^{n}E_{i} \end{array}$$ (28)

Using the procedure described above, the energy dissipated at each control-deflection level was calculated sequentially and then cumulatively summed. The resulting cumulative energy dissipation values are presented in Table 14. Greater cumulative energy dissipation indicates that the specimen dissipated more energy through cracking, plastic deformation, and material damage under the adopted cyclic loading protocol.

**Table 14:** Cumulative energy dissipation of the specimens (kN ∙ mm).

Control-deflection level (mm)	BG1	BG2	BK	BG1/BK	BG2/BK
±2	208.81	285.56	115.74	1.80	2.47
±4	769.63	854.91	336.14	2.29	2.54
±8	3649.41	3980.13	1468.30	2.49	2.71
±16	13941.05	14910.18	5989.31	2.33	2.49
±32	42344.14	44870.72	18587.36	2.28	2.41
+64	65333.44	70223.13	28296.31	2.31	2.48

As shown in Table 14, with the sole exception of BG1 during the first loading cycle, for which the cumulative energy dissipation was 1.80 times that of BK, the values for BG1 and BG2 were more than twice that of BK at all other control-deflection levels. At the end of the loading program, the ratios of the cumulative energy dissipation of BG1 and BG2 to that of BK were 2.31 and 2.48, respectively. Together with the ductility analysis presented above, these results indicate that the U-shaped RC jacket effectively enhanced the energy dissipation capacity of the beams without altering their ductile failure mode, confirming the effectiveness of the adopted strengthening scheme.

### 4.4 Roles of strengthening components and interfacial composite action

The U-shaped RC jacket is composed primarily of jacket concrete, longitudinal reinforcing bars, and stirrups, which work together within the strengthening scheme. By enlarging the cross-section, the jacket increases the effective depth and moment of inertia of the beam, as well as the internal lever arm of the longitudinal reinforcement. The jacket concrete increases the cracking load and contributes to compression under negative loading. After cracking, the added longitudinal reinforcing bars primarily resist tensile forces, thereby increasing the yield load, peak load, and post-yield stiffness; however, an excessively high reinforcement ratio may reduce beam ductility. The added stirrups restrain crack development, enhance shear capacity, and confine the jacket concrete.

The various components can work together only when shear is effectively transferred and relative slip is restrained at the interface between the existing beam and the jacket concrete. According to reference [24], increasing the contact area and restraint through a three-sided U-shaped jacket reduced the maximum interfacial slip to approximately 47% of that obtained with a single-sided jacket; reference [25] reported that surface roughening improved interfacial frictional resistance and stiffness.

In the present study, the interfaces of both strengthened beams were cleaned and coated with a bonding agent, and D6 L-shaped anchor bars were installed across the interfaces. The interface of BG1 was additionally roughened to a depth of approximately 8 mm. Neither BG1 nor BG2 exhibited visible interfacial separation, and their comparable peak loads suggested that the existing concrete and jacket concrete largely acted together under the experimental conditions. The shear-cracking loads of BG1 under positive and negative loading were 251.55 kN and −263.17 kN, respectively, compared with 167.85 kN and −202.47 kN for BG2. In terms of absolute values, the BG1 results were 49.9% and 30.0% higher, respectively, tentatively suggesting that surface roughening may delay the development of shear cracks.

However, because the strengthened specimens differed from BK simultaneously in cross-sectional dimensions, concrete properties, and reinforcement, the tests captured only the overall effect of the U-shaped RC jacket and could not isolate the contributions of individual components. Furthermore, because interfacial slip was not measured, localized microcracking or microslip cannot be ruled out, and further verification through single-parameter tests is required.

### 4.5 Comparison with recent studies

Table 15 summarizes selected recent studies on RC beam strengthening and their key findings.

**Table 15:** Recent studies on RC beam strengthening and their key findings.

Reference	Object of study	Increase in structural capacity	Other performance changes	Conclusion
Li et al. [17] (2022)	HDC jackets	13.6%–145.5%	Improved crack distribution	Local debonding highlights the need to control jacket geometry and interfacial stress concentrations
Ke et al. [19] (2023)	UHPFRC jacket	2.7 ~ 3.0 times	--	Jacket stirrups improve the shear resistance and failure behavior of UHPFRC-strengthened beams.failure behavior
Hong et al. [18] (2023)	UHPC jacket	35% (side) 88% (U-shaped)	21% ~ 61% (ductility), 9% ~ 77% (stiffness)	U-shaped jackets outperformed side jackets, improving load-carrying capacity, ductility, stiffness, and failure behavior
Dar et al. [1] (2024)	RC- FRP jacket	63%, 86%, 108%	90% (stiffness)	The reported improvements reflect the combined effects of the RC jacket and FRP
Zhou et al. [26](2025)	UHPC layers	20.3% ~ 32.2%	--	Surface roughening and post-installed bars enhanced composite action, while the side-applied UHPC layers mainly improved shear resistance
Nugroho et al. [4] (2025)	UHPC layer	85.43%, 119.96%	Increased stiffness and toughness	Increasing longitudinal reinforcement enhanced ultimate load, stiffness, and toughness but slightly reduced ductility

The strengthening schemes employing high-performance materials in Table 15 offer the advantages of thin strengthening layers, high strength, and effective crack control. However, they generally entail high material costs, stringent construction quality-control requirements, and a risk of interfacial debonding. Moreover, most relevant studies employed monotonic loading, leaving the response of such strengthened beams under cyclic loading insufficiently investigated.

To address this gap, cyclic loading experiments were conducted on T-shaped RC beams strengthened with U-shaped RC jackets made from readily available conventional materials. The experimental results showed that the loads of the strengthened beams at the maximum control deflections, their secant stiffnesses, and their final cumulative energy dissipation values were 1.85 ~ 2.67, 2.2 ~ 2.7, and 2.31 ~ 2.48 times those of the existing beam BK, respectively. Neither strengthened beam exhibited visible interfacial separation, and both failed in a ductile manner. These results suggest that, within the investigated parameter range, the strengthening scheme improved the load-carrying capacity, stiffness, and energy dissipation capacity of the beams, with overall trends broadly consistent with those reported in Table 15.

Taken together, the present results and the studies summarized in Table 15 show that RC beams are commonly strengthened by applying cementitious or FRP-based layers or jackets to the soffit, sides, or both. Reinforcing bars may be incorporated as required to promote composite action between the added components and the existing beam. These approaches generally improve load-carrying capacity, stiffness, and crack control. However, the reported performance gains vary considerably because of differences in beam type, initial damage level, strengthening material and thickness, strengthening configuration, and loading protocol.

Accordingly, the relative merits of different strengthening methods should not be assessed solely from the magnitude of the reported performance gains without considering the specific experimental conditions. Moreover, because the present study included relatively few specimens, the applicability and generalizability of its findings require further verification by expanding the sample size and conducting controlled single-parameter experiments.

### 4.6 Engineering applications and scope of applicability

The proposed method is primarily applicable to precast or detachable simply supported RC T-beams seated on bearings, cap beams, or corbels, provided that safe lifting and subsequent reinstallation are feasible. After being removed from their original positions, the beams can undergo interface preparation, jacket casting, and curing under unloaded conditions and in a controlled environment before being returned to service. Accordingly, potential applications include simply supported T-beams in existing industrial and prefabricated buildings, as well as precast T-girders in highway and urban bridges.

Before practical implementation, a comprehensive assessment should be conducted of the existing beam condition, including damage and reinforcement corrosion; the interface condition; the load-carrying capacities of the supports and foundations; and the feasibility of lifting and traffic management. The effects of additional self-weight, enlarged cross-sectional dimensions, and, for bridges, any resulting reduction in under-bridge clearance should also be evaluated. The findings of this study should not be directly extrapolated to continuous or prestressed members cast monolithically with floor slabs or piers, or to beams for which reliable interface preparation cannot be achieved. Applications to such cases require project-specific experimental verification and global structural analysis.

## 5. Finite element analysis

Finite element simulations were performed using ABAQUS. The model comprised an RC beam, loading plates, and supports. The total simulated load was calculated as the sum of the forces transmitted through the loading plates. The loading plates and supports were modeled as steel with an elastic modulus of 210 GPa and a Poisson’s ratio of 0.3. Appropriate constitutive models for the reinforcing bars and concrete were critical to ensuring simulation accuracy.

### 5.1 Material constitutive models

The concrete damage plasticity (CDP) model implemented in ABAQUS was used to represent the constitutive behavior of concrete [43]. The five principal CDP parameters adopted in the analysis are listed in Table 16 [44–47].

**Table 16:** Basic parameters of the CDP model.

Dilation Angle	Eccentricity	fb0/fc0	K	Viscosity Parameter
30	0.1	1.16	0.666667	0.005

The uniaxial compressive and tensile stress–strain relationships and their associated parameters were determined using the concrete constitutive model provided in the appendix to the *Code for Design of Concrete Structures* [12, 33]. The Poisson’s ratio of concrete was taken as 0.2, and the adopted stress–strain relationships are shown in Fig 12. The parameters *α*_c_ and *α*_t_ govern the descending branches of the compressive and tensile stress-strain curves, respectively. The value of *α*_c_ increases with concrete compressive strength, whereas *α*_t_ increases with concrete tensile strength; the adopted values are listed in Table 17.

**Table 17:** Values of the concrete constitutive parameters.

Concrete types	*f*_c,r_ (N/mm^2^)	*f*_t,r_ (N/mm2)	*ε*_c,r_ (%)	*ε*_t,r_ (10^−6^)	*α*_c_	*α*_t_
Existing beam	13.68	2.1	0.17	97.4	0.35	1.390
Strengthened portion	26.45	2.9	0.23	115.8	0.90	2.638

**Fig 12:**
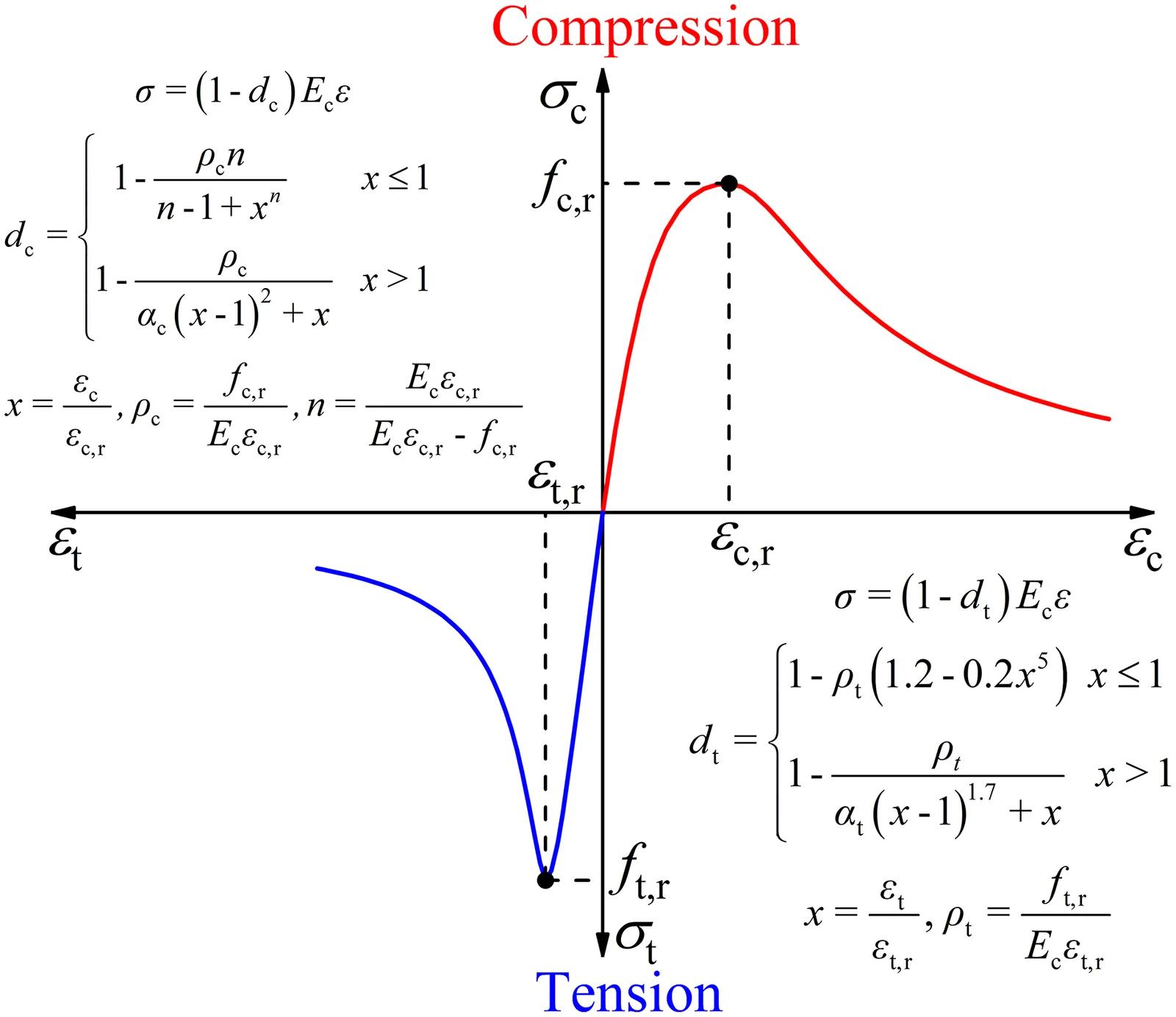
Concrete constitutive curves (different scales).

Owing to the concrete size effect, compressive strength decreases with increasing member size [48]. This behavior is generally attributed to inherent defects and discontinuities within the concrete [49]. To account for this effect, reduced compressive strengths were adopted in the finite element analysis. The values of fc,r listed in Table 17 are the reduced compressive strengths determined in accordance with ACI 318−19 [50].

The steel loading plates and supports were modeled as linearly elastic. The reinforcing bars were modeled using a bilinear constitutive relationship, with a Poisson’s ratio of 0.3. The yield point divides the constitutive curve into pre- and post-yield linear branches [51,52], as shown in Fig 13.

**Fig 13:**
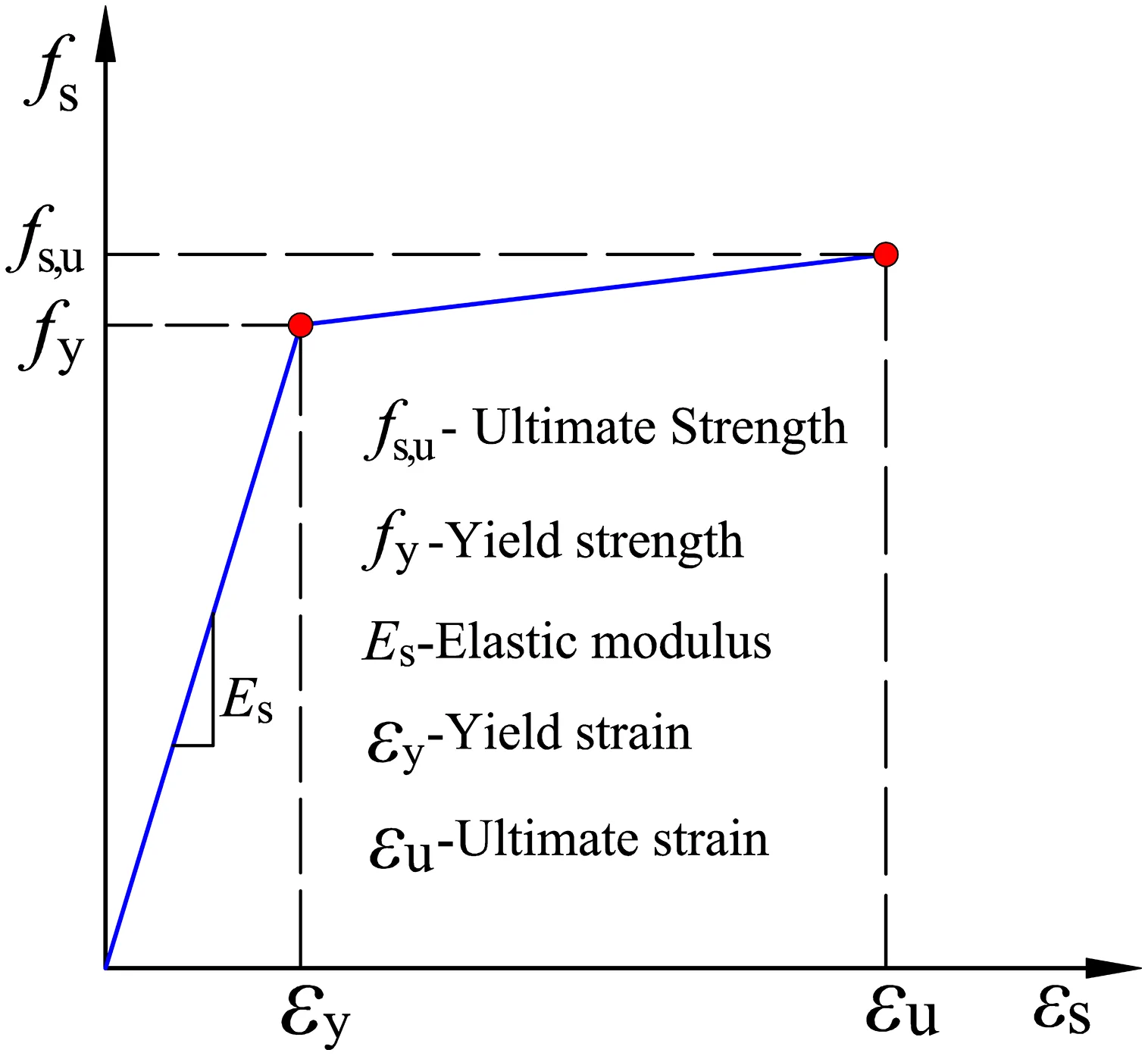
Stress–strain curve for reinforcing bars.

### 5.2 Finite element model

Fig 14 presents the finite element model together with its boundary conditions and interaction definitions. Identical loading plates and supports were arranged on the upper and lower faces of the RC beam to reproduce the load reversal adopted in the experiments. Reference points RP1 ~ RP4 were coupled to the upper surfaces of the corresponding loading plates and used as loading control points [52].

**Fig 14:**
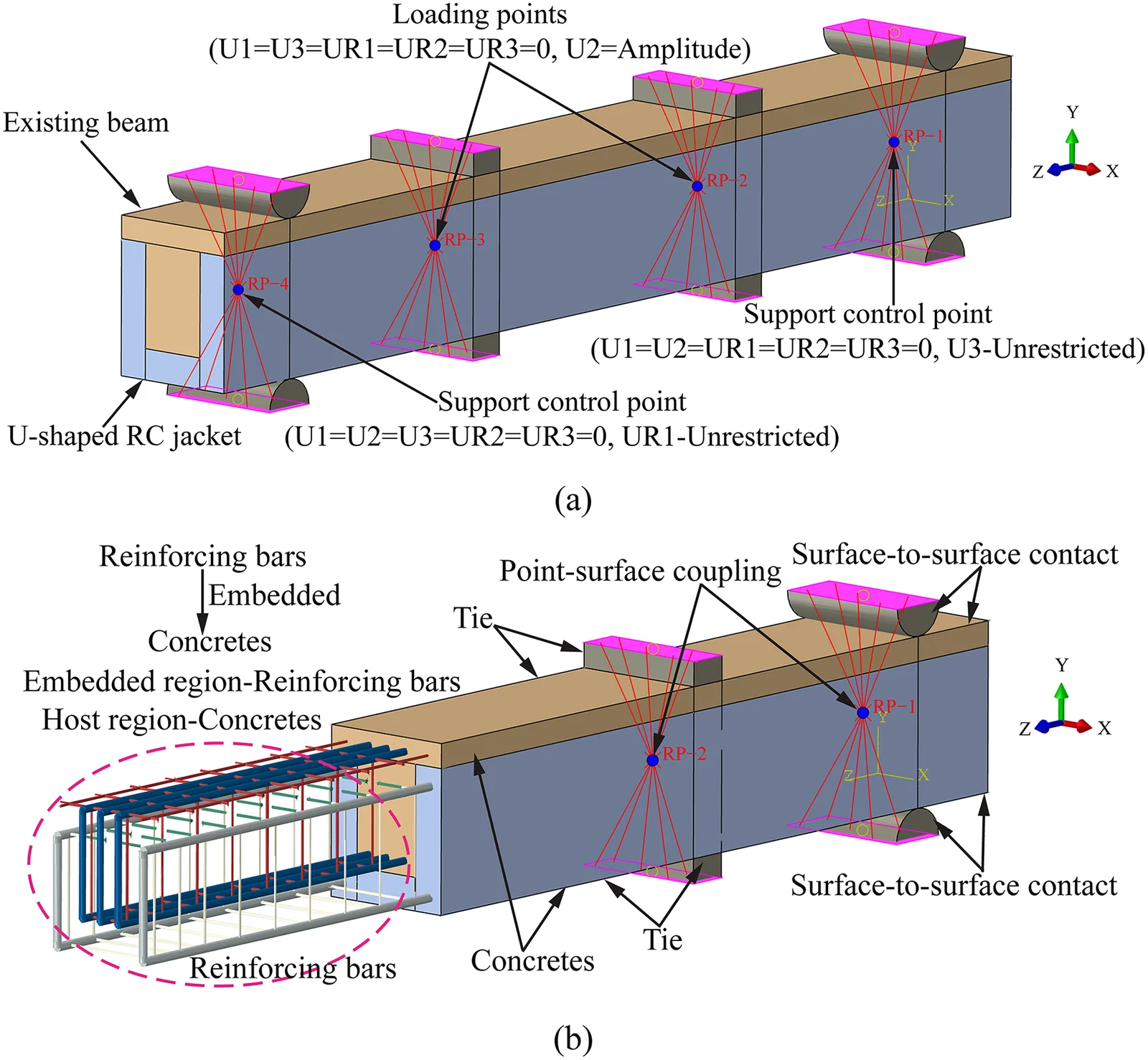
3D finite element model. (a) Boundary conditions. (b) Interactions.

The interaction between the steel supports and the RC beam was modeled using surface-to-surface contact [12]. Hard contact was specified in the normal direction, whereas relative sliding in the tangential direction was represented using a friction coefficient of 0.5 [53]. Bond-slip behavior between the reinforcing bars and concrete was modeled using the Embedded Region constraint in conjunction with a bond-slip subroutine [54]. Tie constraints were used between the loading plates and the beam.

The preceding experimental results tentatively suggested that surface roughening improved the interface behavior of BG1 relative to BG2. Nevertheless, neither strengthened specimen exhibited visible macroscopic separation or extensive debonding at the interface between the existing and jacket concrete, and both components largely acted as an integrated whole. The interfaces were therefore idealized as fully bonded for simulating the global response.

To distinguish the two interface treatments numerically, coincident interface nodes were merged in BG1, whereas a Tie constraint was applied between the independently meshed surfaces in BG2. Both approaches suppress explicit interface separation and slip and should not be interpreted as calibrated representations of different bond strengths. Consequently, this idealization does not capture localized microcracking, microslip, or interfacial damage.

The concrete, loading plates, and supports were discretized using C3D8R elements (eight-node linear brick elements with reduced integration and hourglass control), whereas the reinforcing bars were modeled using T3D2 elements (two-node linear three-dimensional truss elements) [45,52,55]. A target mesh size of 30 mm was adopted for the concrete and reinforcing bars.

### 5.3 Finite element analysis results

#### 5.3.1 Load-deflection response.

The experimental and simulated hysteresis and skeleton curves of each specimen are compared in Figs 15 and 16, respectively. The simulated loads are alternately higher and lower than the experimental values at different loading stages. These discrepancies may be attributed to the omission of material heterogeneity and inherent specimen defects, idealized interactions and boundary conditions, and simplified modeling of the new-to-old concrete interface. Nevertheless, both the experimental and simulated responses exhibit progressive stiffness degradation and broadly consistent overall trends, indicating that the FE model can reasonably reproduce the global hysteretic behavior of the RC beams.

**Fig 15:**
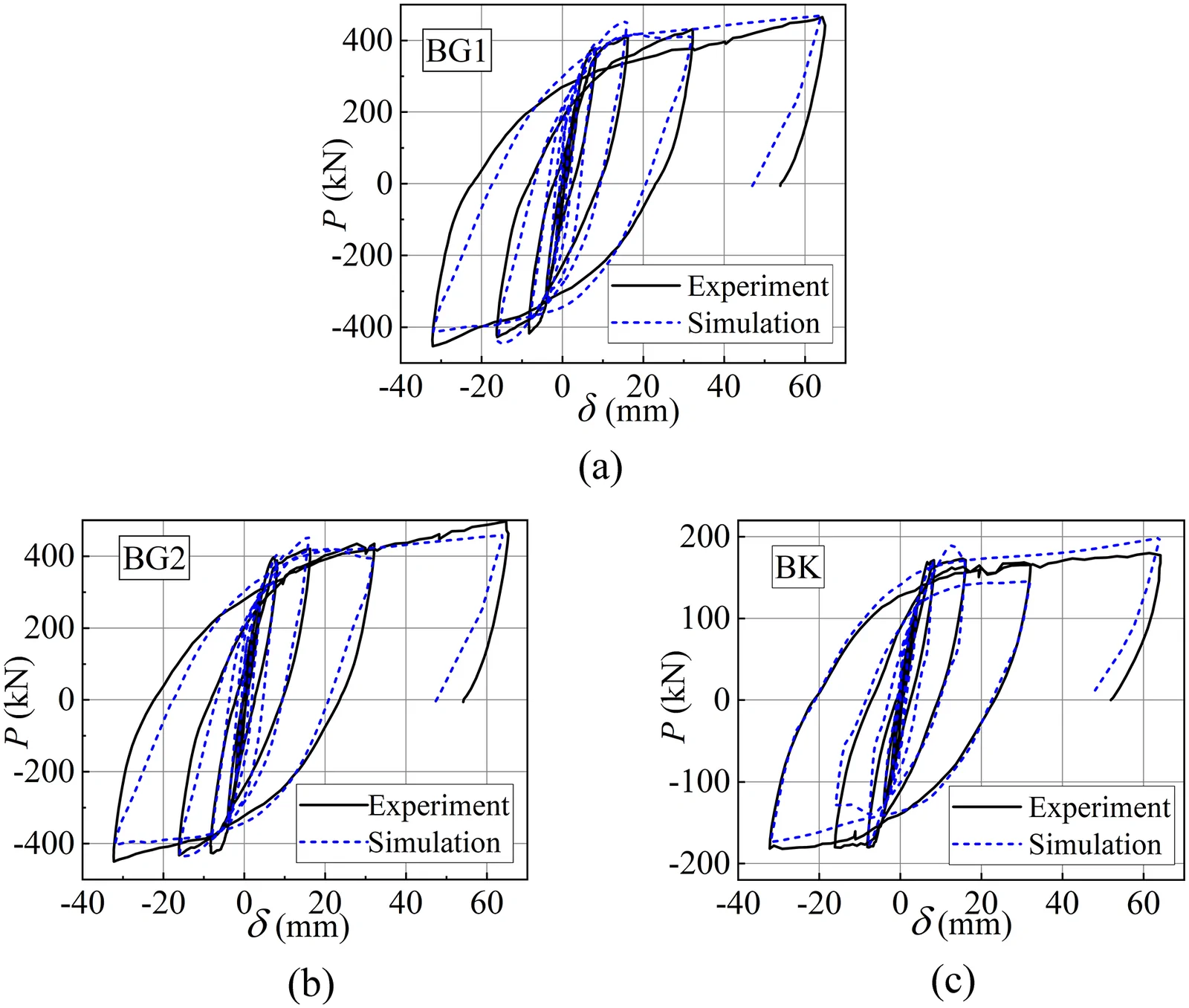
Experimental and simulated hysteresis curves. (a) BG1. (b) BG2. (c) BK.

**Fig 16:**
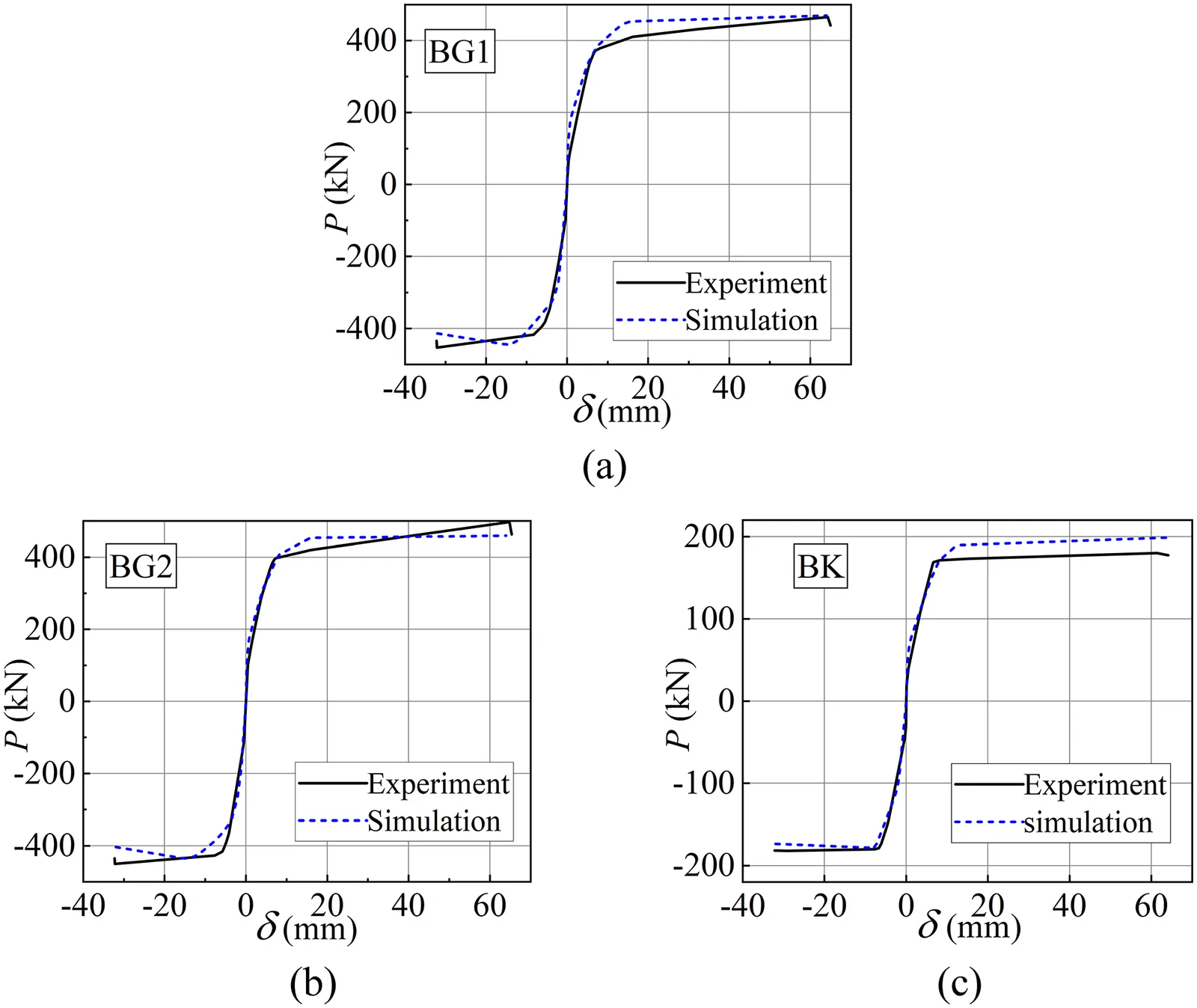
Experimental and simulated skeleton curves. (a) BG1. (b) BG2. (c) BK.

#### 5.3.2 Quantitative validation of the finite element model.

To quantitatively assess the accuracy of the finite element model, the experimental and simulated skeleton and hysteresis curves were compared using common deflection grids. For each loading direction of the skeleton curves, 101 equally spaced deflection points were selected within the common experimental–numerical range, and the corresponding loads were obtained by piecewise-linear interpolation. For the hysteresis curves, the loading history was divided into 12 matching branches according to the control-deflection amplitudes, and each branch was resampled at 51 equally spaced points within its common deflection range.

Curve agreement was quantified using the mean absolute error (MAE), root mean square error (RMSE), normalized root mean square error (NRMSE), and coefficient of determination (*R*²). The NRMSE was calculated by normalizing the RMSE by the maximum absolute experimental load within the corresponding comparison range. The percentage errors in the positive and negative peak loads were also calculated. Let $P_{i}^{FE}$ and $P_{i}^{Exp}$ denote the simulated and experimental loads, respectively, at the *i* th aligned deflection point, and let *n* denote the number of aligned points. The error metrics are calculated as follows:


$$\begin{array}{c} \text{MAE\textasciitilde{}=\textasciitilde{}}\frac{\text{1}}{n}\sum_{i\text{=}1}^{n}|P_{i}^{FE}\text{-}P_{i}^{Exp}|\; \end{array}$$ (29)

$$\begin{array}{c} \text{RMSE=}\sqrt{\frac{\sum_{i\text{=}1}^{n}(P_{i}^{FE}\text{-}P_{i}^{Exp}{)}^{\text{2}}}{n}} \end{array}$$ (30)

$$\begin{array}{c} \text{NRMSE=}\frac{RMSE}{\text{max}\left|P_{i}^{Exp}\right|}\times \text{100\textbackslash{}\%} \end{array}$$ (31)

$$\begin{array}{c} R^{\text{2}}\text{=1-}\frac{\sum_{i\text{=}1}^{n}(P_{i}^{FE}-P_{i}^{Exp}{)}^{\text{2}}}{\sum_{i\text{=}1}^{n}(P_{i}^{Exp}\text{-}{\overset{―}{P}}^{Exp}{)}^{\text{2}}} \end{array}$$ (32)

MAE and RMSE are expressed in kN and quantify the mean error magnitude and sensitivity to larger deviations, respectively. NRMSE is normalized by the maximum absolute experimental load within the corresponding comparison range, enabling comparisons among specimens with different load-carrying capacities. The coefficient of determination, *R*², quantifies the overall goodness of fit between the experimental and simulated curves. The percentage error in each positive or negative peak load, *e*_p_, is calculated using Eq 33:


$$\begin{array}{c} e_{p}\text{=}\frac{\left|P_{p}^{FE}\text{-}P_{p}^{Exp}\right|}{\left|P_{p}^{Exp}\right|}\times 100\%\; \end{array}$$ (33)

To distinguish the main cyclic response from the final unloading response, the hysteresis curve was divided into 12 branches. The first 11 branches constitute the main hysteresis path, whereas the full path includes all 12 branches. The final unloading path corresponds to the 12th branch, extending from +64 mm to the unloading endpoint. The resulting validation metrics for the skeleton and hysteresis curves are summarized in Tables 18–20.

**Table 18:** Overall error metrics and peak-load errors for the skeleton curves.

Specimen	MAE (kN)	RMSE (kN)	NRMSE (%)	*R*²	Positive peak-load error (%)	Negative peak-load error (%)
BK	11.07	12.80	7.03	0.994	10.32	1.95
BG1	21.83	25.88	5.56	0.996	0.93	1.88
BG2	20.30	24.35	4.91	0.997	7.68	3.39

**Table 19:** Quantitative validation results for the main hysteresis paths.

Specimen	MAE (kN)	RMSE (kN)	NRMSE (%)	*R*²	Positive-branch NRMSE (%)	Negative branch NRMSE (%)
BK	17.24	21.12	11.63	0.959	10.48	12.99
BG1	55.23	67.68	14.57	0.919	14.89	14.56
BG2	46.59	62.12	12.53	0.940	11.59	14.98

**Table 20:** Quantitative validation results for the full hysteresis paths and final unloading branches.

Specimen	Full hysteresis path				Final unloading NRMSE (%)
	MAE (kN)	RMSE (kN)	NRMSE (%)	*R*²	
BK	19.68	24.31	13.39	0.943	28.41
BG1	62.86	77.49	16.69	0.888	40.64
BG2	55.29	73.80	14.89	0.909	43.77

Table 18 shows that the NRMSE values for the skeleton curves of BK, BG1, and BG2 were 7.03%, 5.56%, and 4.91%, respectively, all below 10%. The corresponding *R*² values were 0.994, 0.996, and 0.997. The positive and negative peak-load errors were 0.93% ~ 10.32% and 1.88% ~ 3.39%, respectively. These results indicate that the finite element model accurately reproduces the overall trends and peak responses of the experimental skeleton curves.

Table 19 shows that the MAE values for the main hysteresis paths of BK, BG1, and BG2 were 17.24 kN, 55.23 kN, and 46.59 kN, respectively, while the corresponding RMSE values were 21.12 kN, 67.68 kN, and 62.12 kN. The NRMSE values were 11.63%, 14.57%, and 12.53%, and the *R*² values were 0.959, 0.919, and 0.940, respectively. These results indicate that the finite element model reasonably reproduces the evolution of load-carrying capacity and the overall trend of stiffness degradation under cyclic loading.

Table 20 shows that, when the final unloading branch was included, the NRMSE values for BK, BG1, and BG2 increased to 13.39%, 16.69%, and 14.89%, respectively, while the corresponding *R*² values decreased to 0.943, 0.888, and 0.909. For the final unloading branch alone, the NRMSE values were 28.41%, 40.64%, and 43.77%, indicating that the model reproduced the final unloading stiffness, unloading endpoint, and residual deformation less accurately than the main hysteresis path. The larger errors may be attributed to accumulated cracking, contact gaps, and potential interfacial microslip during the later loading stages, which were not fully represented by the idealized finite element model.

Based on the quantitative comparisons of the skeleton curves, peak loads, and hysteresis paths, the developed finite element model reasonably reproduces the global load-carrying capacity, skeleton response, and main hysteresis paths of specimens BK, BG1, and BG2. Within the validated parameter range, the model can be used to evaluate the global structural response of unstrengthened and U-shaped RC-jacketed beams and to support subsequent parametric analyses of the CDP viscosity parameter and jacket longitudinal-bar diameter. However, its accuracy in reproducing the final unloading branch is limited; therefore, results concerning unloading stiffness, residual deflection, and detailed energy-dissipation distribution should be interpreted with caution.

#### 5.3.3 Failure modes of RC beams.

As the number of loading cycles and the imposed deflection amplitude increased, the cracks became more numerous, wider, and longer, and the longitudinal reinforcing bars eventually yielded. With accumulating tensile damage, the contribution of the concrete in the tension zone diminished, causing the longitudinal reinforcing bars to carry a greater proportion of the tensile force. As shown in Figs 17(a) and 17(b), the dominant cracks progressively widened, eventually causing localized concrete spalling.

**Fig 17:**
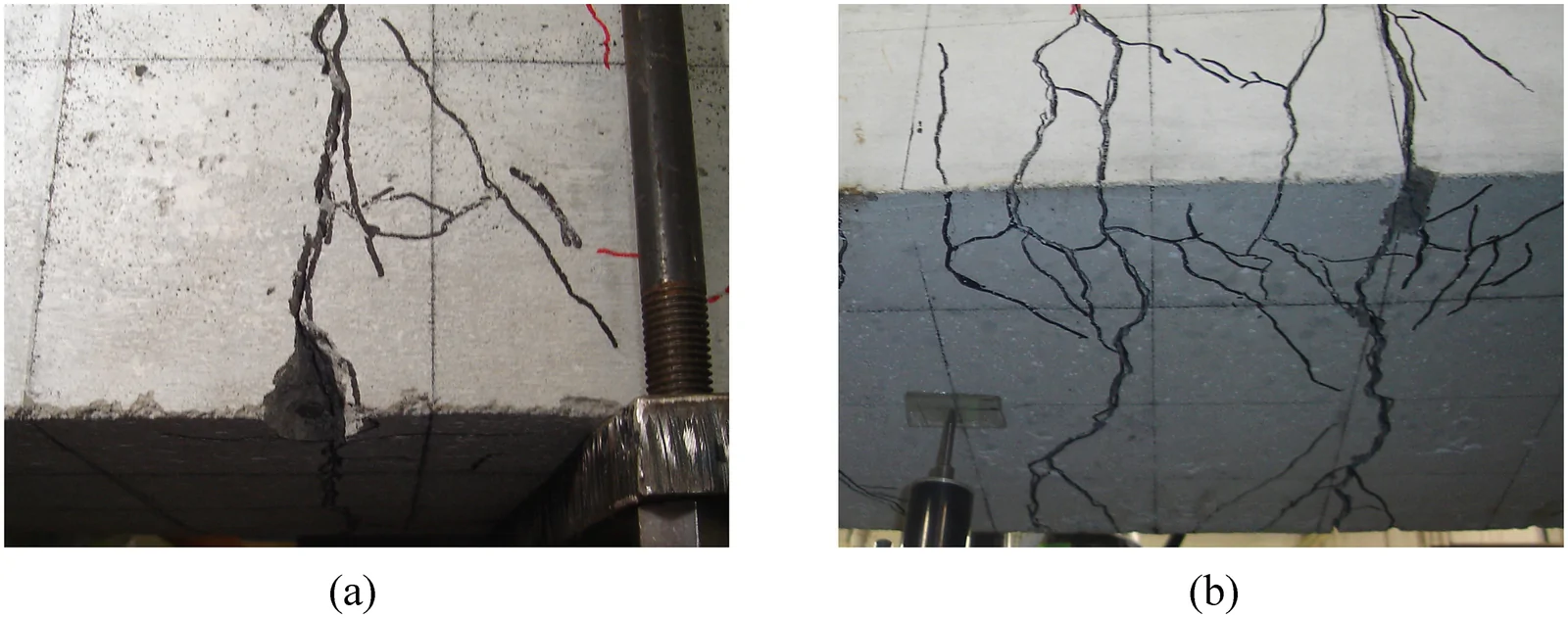
Localized concrete spalling. (a) Location 1. (b) Location 2.

Figs 18–20 compare the experimentally observed and numerically simulated failure modes. The experiments showed that cracking was concentrated mainly within the span between the supports. Shear cracks predominated in the shear spans between the supports and loading plates, whereas flexural cracks were concentrated in the constant-moment region between the loading points. Shear cracking was more pronounced in BG1 and BG2 than in BK.

**Fig 18:**
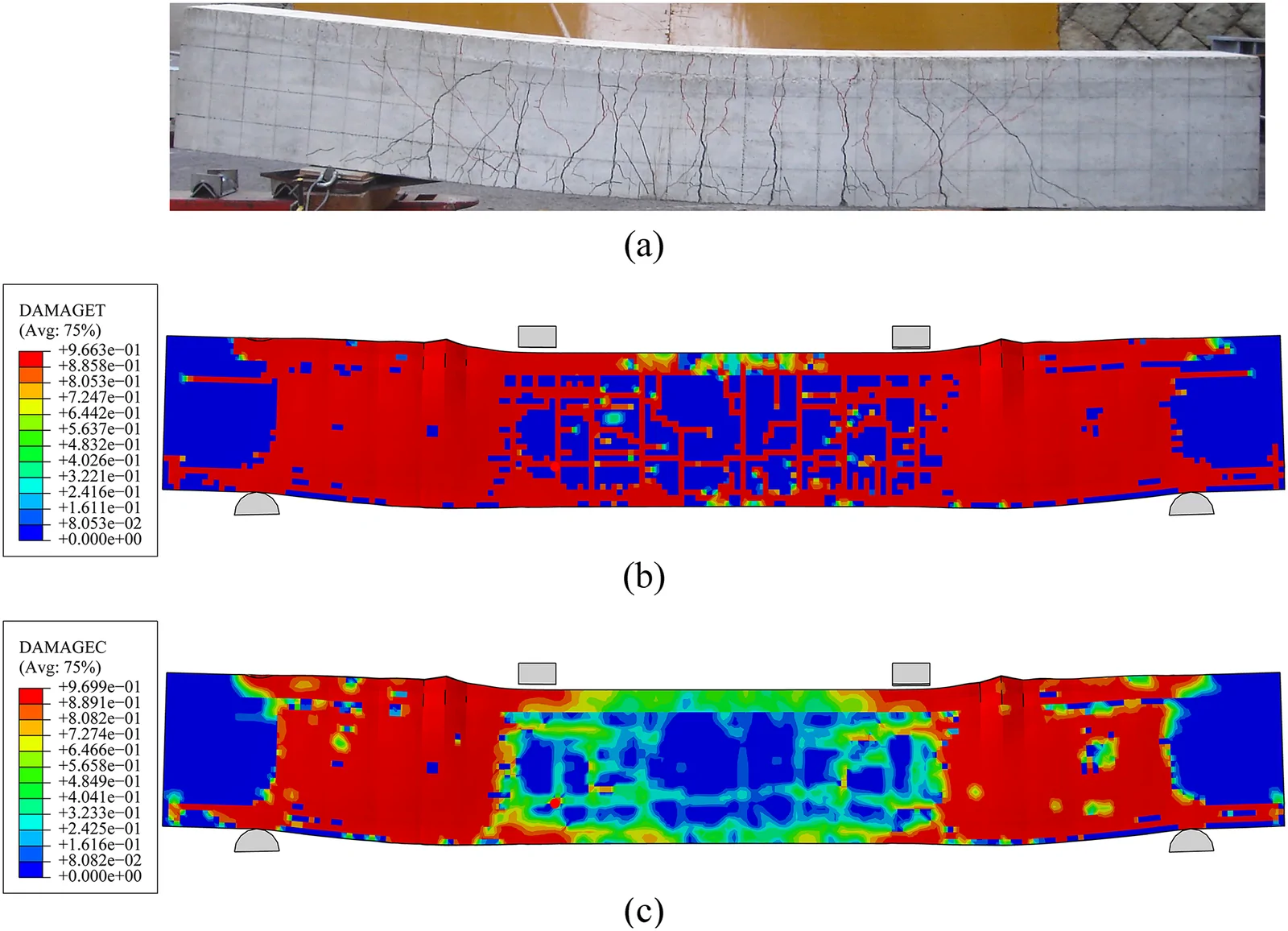
Failure modes of BG1. (a) Experiment. (b) Damage T. (c) Damage C.

**Fig 19:**
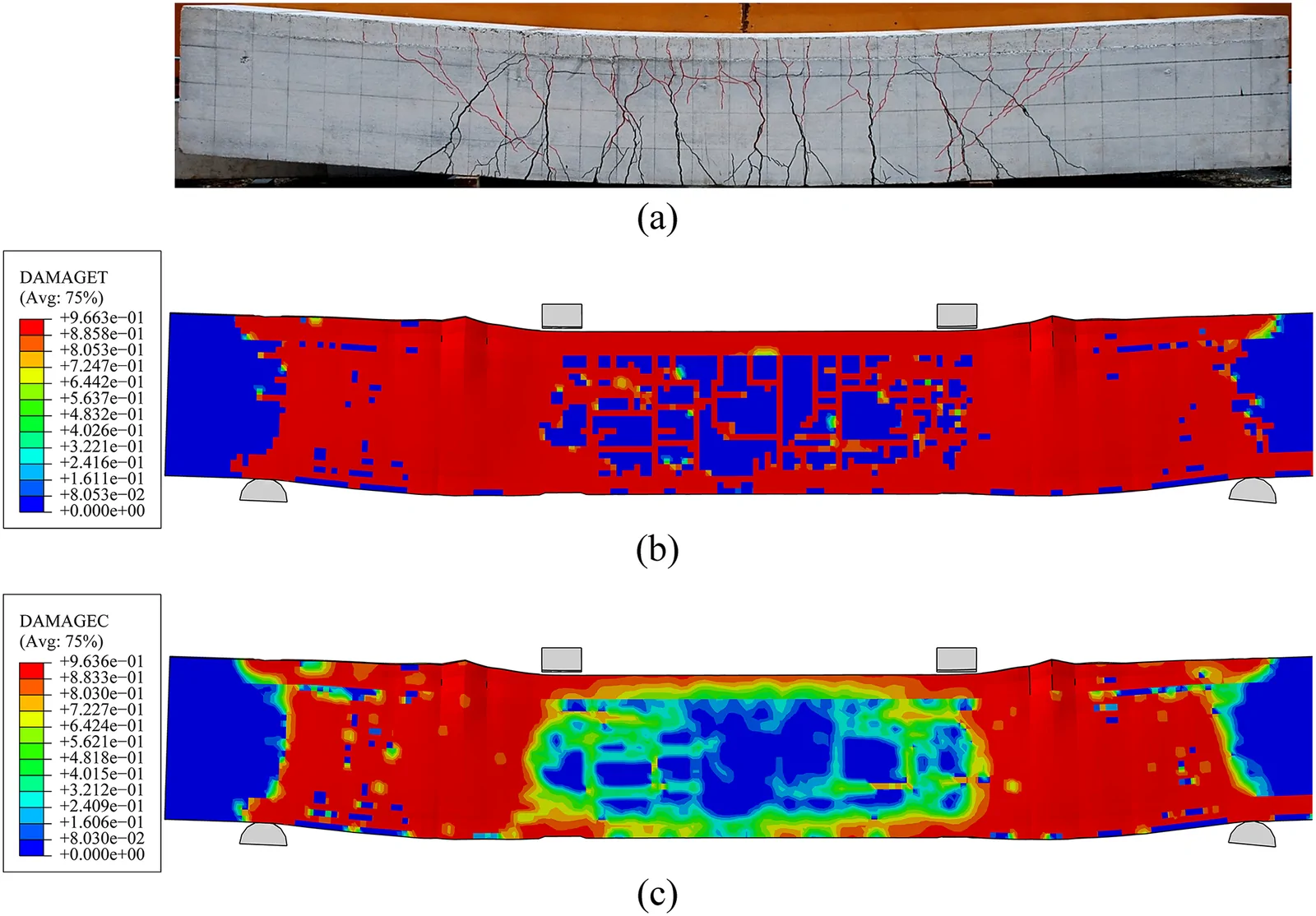
Failure modes of BG2. (a) Experiment. (b) Damage T. (c) Damage C.

**Fig 20:**
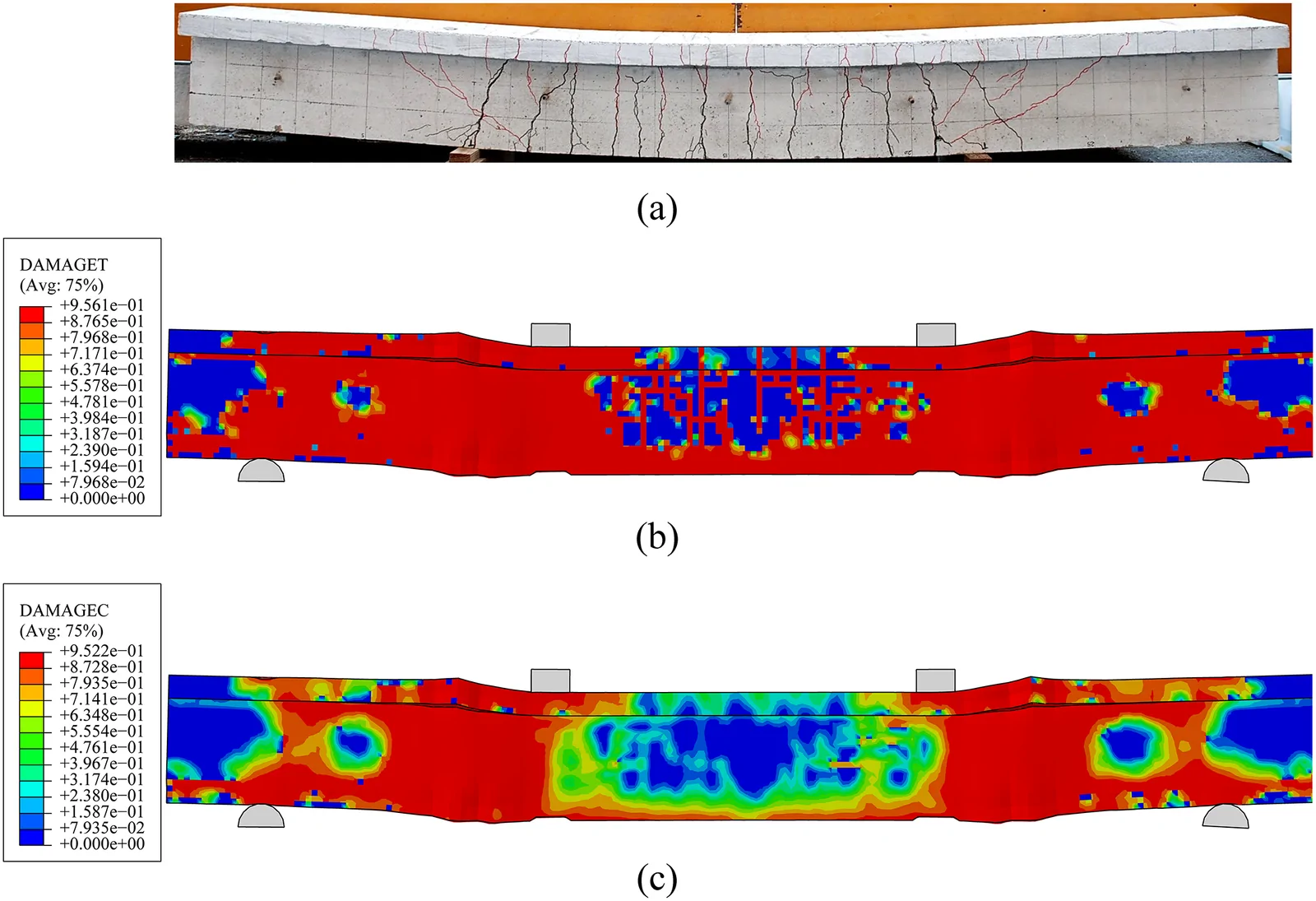
Failure modes of BK. (a) Experiment. (b) Damage T. (c) Damage C.

The simulation results show that the shear spans between the supports and loading points, which were subjected to combined bending and shear, developed substantial tensile and compressive damage. In the constant-moment region between the loading plates, where flexure dominated, the overall damage was less severe, and tensile damage was more pronounced than compressive damage. Overall, the spatial distribution of the simulated damage agreed well with the experimentally observed crack patterns, indicating that the finite element model reasonably reproduced the failure modes of the beams.

### 5.4 Effects of the viscosity parameter and main reinforcing-bar diameter

Based on the finite element model of BG1, this section investigates the influence of the viscosity parameter on the simulated response and optimizes the diameter of the upper longitudinal reinforcing bars in the strengthening jacket.

#### 5.4.1 Viscosity parameter (*V*p).

Figs 21 and 22 show that the simulated load levels increased with *V*p, particularly within the blue dashed regions. As *V*p increased, the hysteresis curves became smoother and fuller, with fewer numerical oscillations and discontinuities. The computation time also decreased, indicating improved convergence. As shown in Fig 21(a), a small *V*p value resulted in an incomplete hysteresis curve because the analysis failed to converge and terminated prematurely. Therefore, cases with *V*p were not considered further. For the present model, *V*p values of 0.002 ~ 0.005 provided an appropriate balance between computational efficiency and simulation accuracy.

**Fig 21:**
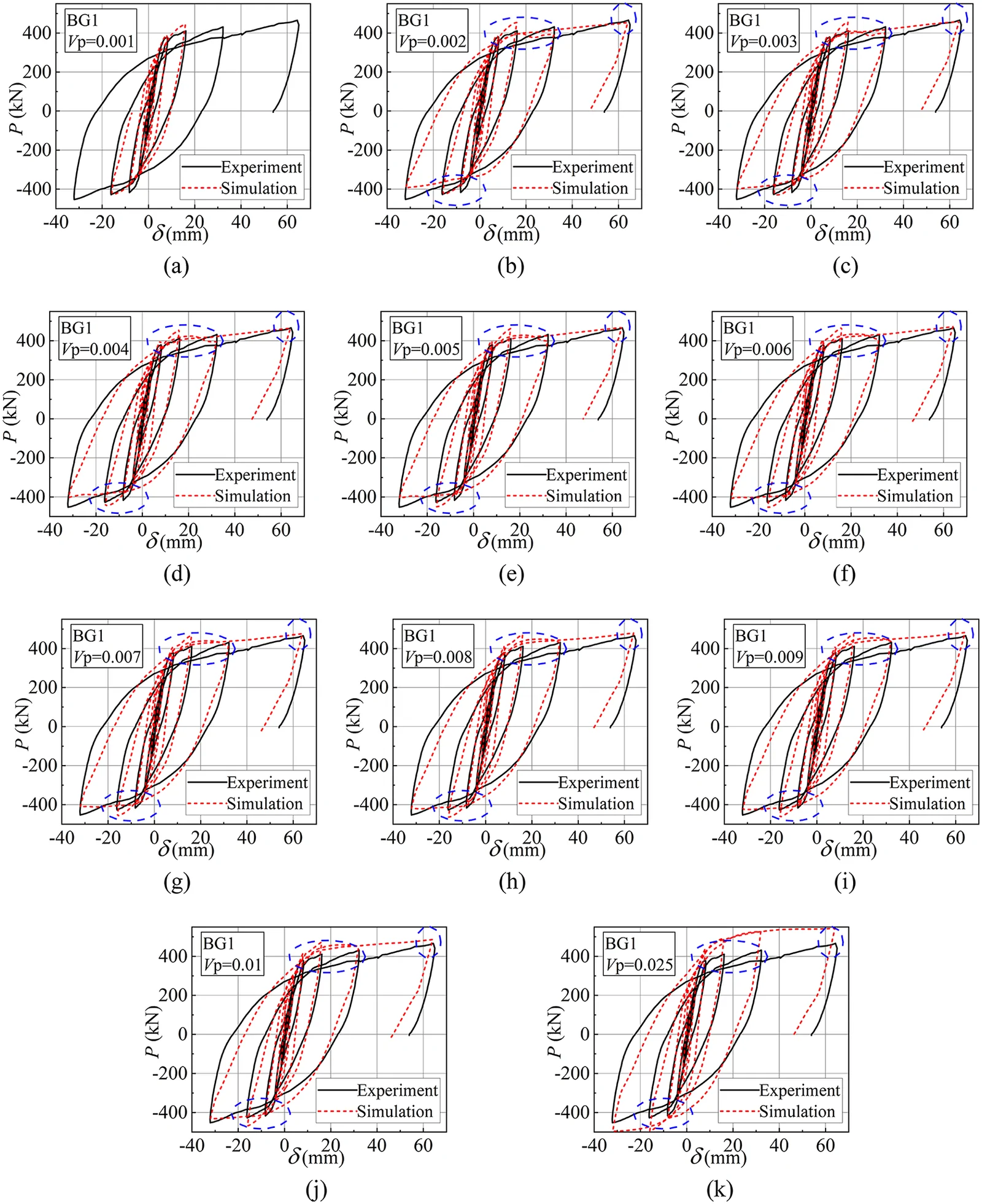
Hysteresis curves for different *V*p values. (a) 0.001. (b) 0.002. (c) 0.003. (d) 0.004. (e) 0.005. (f) 0.006. (g) 0.007. (h) 0.008. (i) 0.009. (j) 0.01. (k) 0.025.

**Fig 22:**
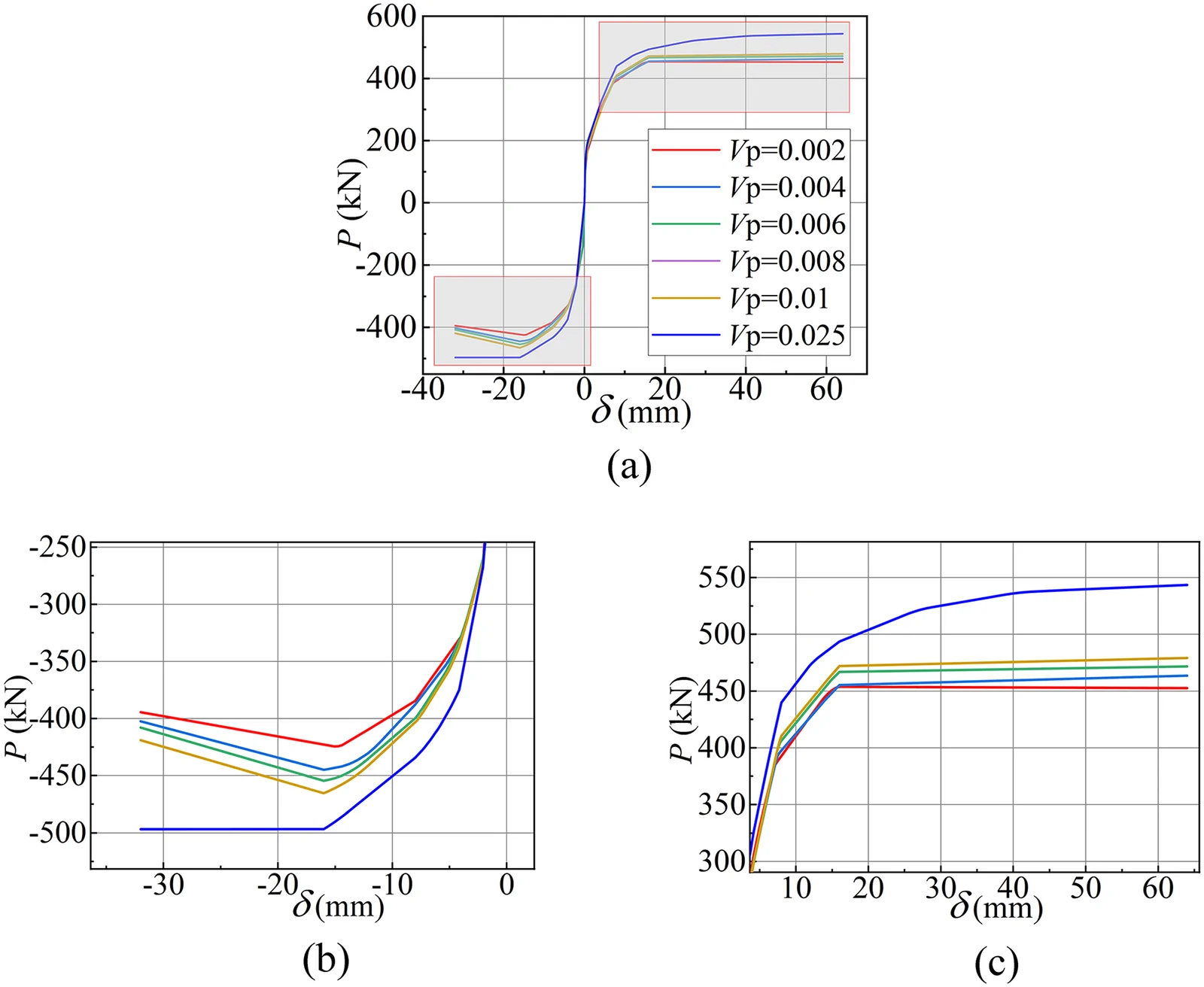
Skeleton curves for different *V*p values. (a) Enlarged view 1. (b) Skeleton curves. (c) Enlarged view 2.

#### 5.4.2 Upper longitudinal reinforcing-bar diameter.

The simply supported RC beams considered in this study are generally subjected to unidirectional bending under service loads, corresponding to positive bending in the present analysis. Therefore, their performance is governed primarily by the positive-direction response. The two D20 longitudinal reinforcing bars in the upper part of the strengthening jacket, adjacent to the flange of the existing T-beam, primarily secure the stirrups and maintain the reinforcement cage rather than provide substantial flexural resistance. Using D20 bars at this location may therefore be inefficient, and smaller-diameter bars of the same grade could reduce material consumption, provided that the relevant detailing requirements are satisfied. Fig 23 compares the simulated hysteresis curves obtained by replacing the D20 bars with D8, D10, D12, and D14 bars.

**Fig 23:**
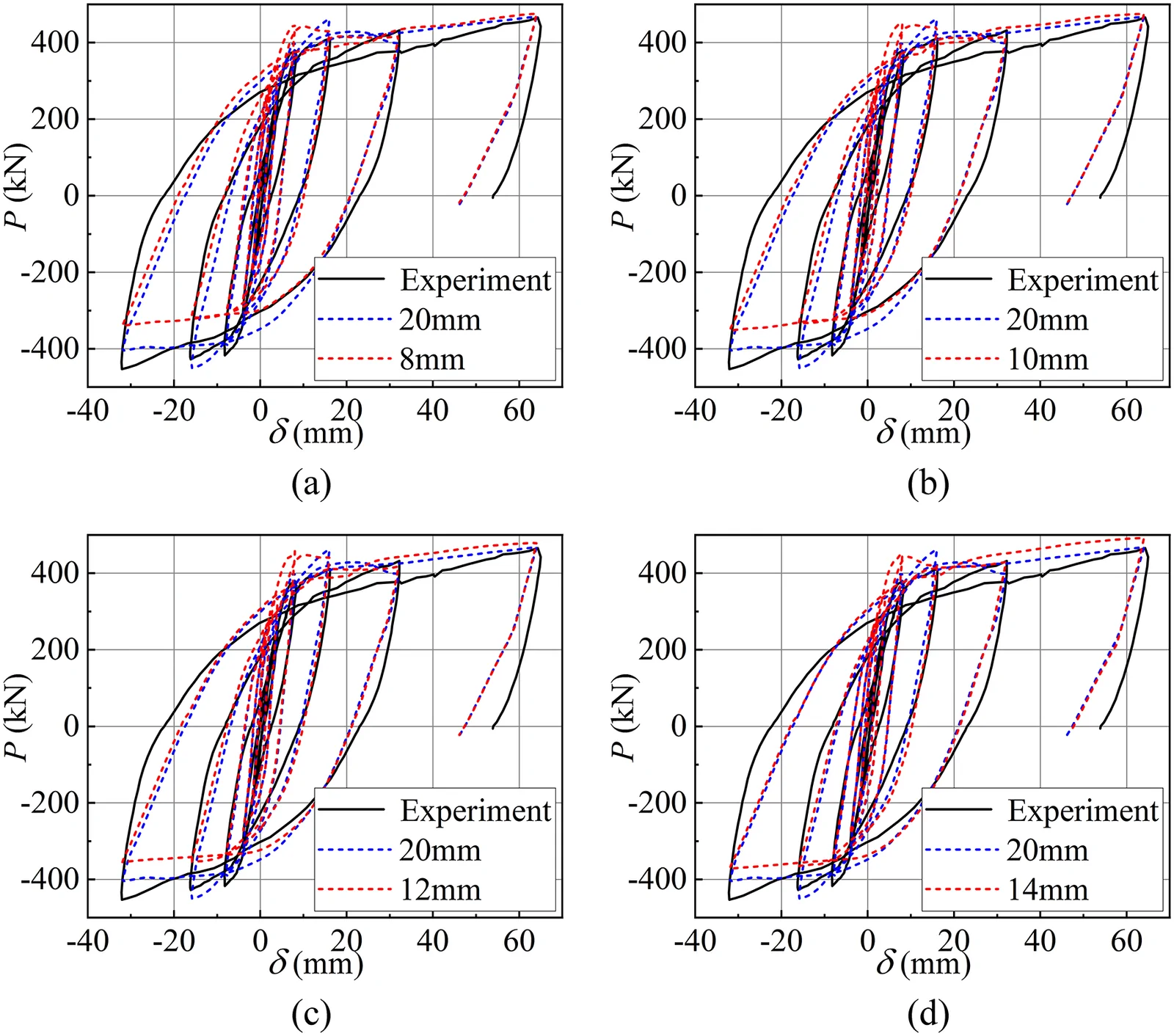
Hysteresis curves for different upper longitudinal reinforcing bar diameters. (a) 8 mm. (b) 10 mm. (c) 12 mm. (d) 14 mm.

As shown in Fig 23, decreasing the diameter of the upper longitudinal reinforcing bars in the strengthening jacket reduced the negative-direction load-carrying capacity and the area enclosed by the negative-loading branches. In contrast, the positive-direction load-carrying capacity and the corresponding enclosed area increased. Nevertheless, all hysteresis curves remained full and exhibited no evident pinching, indicating that the diameter reduction did not alter the overall stiffness degradation pattern or ductile flexural behavior. Within the investigated diameter range, smaller upper longitudinal reinforcing bars could therefore reduce steel consumption without compromising the positive-direction load-carrying capacity. However, the associated reduction in negative-direction capacity should be considered where load reversal or negative bending is relevant.

## 6. Conclusions

This study investigated one unstrengthened simply supported T-shaped RC beam (BK) and two beams strengthened with 80 mm-thick U-shaped RC jackets (BG1 and BG2). Based on quasi-static cyclic loading experiments, theoretical analysis, and finite element simulations, the following main conclusions were drawn:

(1) The 80-mm-thick U-shaped RC jacket converted the original T-section into a rectangular composite section. For BG1 and BG2, the loads corresponding to the maximum deflection control points and the secant stiffnesses were 1.85 ~ 2.67 and 2.2 ~ 2.7 times the corresponding values for BK, respectively. The final cumulative energy dissipation values of BG1 and BG2 were 2.31 and 2.48 times that of BK, respectively. No visible interfacial separation was observed, and both strengthened beams exhibited ductile failure.
(2) Preliminary test results indicate that the positive- and negative-direction shear cracking loads of BG1 with a roughened interface were approximately 49.9% and 30.0% higher than those of BG2, respectively. The comparison between BG1 and BG2 provides preliminary evidence that interface roughening enhances shear transfer and delays diagonal cracking. At the prescribed deflection-control points, the BG2-to-BG1 load ratio reached a maximum of 1.30 and gradually decreased to 1.01 with increasing deflection. However, the influence of interface roughening on the load-carrying capacity requires further verification using additional specimens.
(3) Within the investigated parameter range and under symmetric two-point loading, the truss–arch model predicted the positive- and negative-direction load-carrying capacities of BK, BG1, and BG2 with absolute errors of 0.28% ~ 9.76% and 2.66% ~ 7.92%, respectively. Truss action contributed approximately 83% ~ 89% of the predicted capacity and was the primary load-transfer mechanism. However, owing to the small number of validation specimens and narrow parameter range, the model requires further verification and should currently be regarded only as a supplement to code-based calculation methods.
(4) The finite element model reproduced the skeleton curves and main hysteresis paths of the load–deflection response reasonably well. For the skeleton curves, the NRMSE and *R*^2^ values were 4.91% ~ 7.03% and 0.994 ~ 0.997, respectively, while the positive and negative peak-load errors were 0.93% ~ 10.32% and 1.88% ~ 3.39%, respectively. For the main hysteresis paths, the NRMSE and *R*^2^ values were 11.63% ~ 14.57% and 0.919 ~ 0.959, respectively.
(5) The NRMSE of the final-stage unloading branch further increased to 28.41% ~ 43.77%. For the full hysteretic path including the final-stage unloading branch, the NRMSE increased to 13.39% ~ 16.69%, and R² decreased to 0.888 ~ 0.943. This indicates that the model still needs to be used cautiously when evaluating the late-stage unloading stiffness and the response at the unloading endpoint.
(6) Within the investigated parameter range, the diameter of the upper longitudinal reinforcing bars in the strengthening jacket could be reduced from D20 to D8 ~ D14, provided that the required negative-direction load-carrying capacity and relevant detailing requirements are satisfied. This reduction had little effect on the positive-direction load-carrying behavior or ductile failure mode of the beam and could reduce steel consumption.
(7) By enlarging the section and converting it into a rectangular composite section, U-shaped RC jacketing increases the moment of inertia, internal lever arm, and effective shear area while improving response symmetry under positive and negative loading. This method is primarily applicable to simply supported beams in buildings and bridges that can be safely lifted from their supports, strengthened and cured under unloaded conditions, and subsequently returned to service. Before practical application, the additional self-weight, enlarged cross-sectional dimensions, clearance requirements, and support bearing capacity should be checked.

## 7. Limitations and future work

Only three specimens were tested; therefore, experimental variability could not be adequately assessed. Only one jacket thickness (80 mm) and one reinforcement configuration were examined, and the interface comparison was limited to roughened and unroughened conditions without quantitative measurements of surface roughness or interfacial slip. Moreover, the specimens were simply supported T-beams subjected to quasi-static cyclic loading with asymmetric deflection amplitudes, and the negative-direction loading sequence did not reach the ultimate state.

The theoretical model assumes full composite action between the existing and jacket concrete, whereas the finite element model idealizes the interface using merged nodes for BG1 and Tie constraints for BG2. These idealizations allow the global load–deflection response and load-carrying capacity to be evaluated but cannot explicitly reproduce interfacial damage, relative slip, or debonding. Accordingly, the finite element results are not used to assess local interface behavior, and this interface idealization may partly account for the relatively large errors in the final unloading response.

Future studies should include replicate specimens and systematically investigate the effects of jacket thickness, jacket reinforcement ratio, interface roughness, anchorage configuration, shear-span-to-depth ratio, and pre-existing damage level. Interfacial bond–slip behavior and long-term durability should also be examined. The resulting database could support further refinement and validation of the truss-arch model. Future finite element analyses should incorporate interfacial damage and bond–slip models to improve the simulation of cyclic unloading behavior.

## Supporting information

S1 Data Data. (XLSX)
